# Evaluating the *manubrium sterni* as a site for bone implantation: a 3D radiological feasibility study

**DOI:** 10.1186/s41747-025-00642-6

**Published:** 2025-12-03

**Authors:** Max Johannes Dullaart, Maarten Jan Antony van Alphen, Annelieke Boudina Schoen, Margje Bertine Buitenhuis, Loes Margaretha Marie Braun, Menno Krap, Michiel Wilhelmus Maria van den Brekel, Luc Hendricus Elizabeth Karssemakers, Richard Dirven

**Affiliations:** 1 Department of Head and Neck Oncology and Surgery, Netherlands Cancer Institute, Amsterdam, The Netherlands; 2 Academic Center for Dentistry Amsterdam, Amsterdam, The Netherlands; 3 University Medical Center Utrecht, Utrecht, The Netherlands; 4 Mobius 3D Technologies, Nieuw-Vennep, The Netherlands; 5 Department of Radiology, Netherlands Cancer Institute, Amsterdam, The Netherlands; 6 Foundation of Special Dental Care, Amsterdam, The Netherlands; 7 Amsterdam Center for Language and Communication, University of Amsterdam, Amsterdam, The Netherlands

**Keywords:** Bone implant, Bone morphology, Bone thickness map, Cancellous bone, Total laryngectomy

## Abstract

**Background:**

To increase the number of patients who can speak hands-free after total laryngectomy, we aim to assess the viability of the *manubrium sterni* *bone* (MSB) for bone implantation.

**Materials and methods:**

This retrospective cross-sectional study was conducted at the Department of Head and Neck Surgery of the Netherlands Cancer Institute in Amsterdam, the Netherlands, after approval by its Institutional Review Board. We included CT scans of 25 males and 24 females aged 53 to 88 years, and measured MSB morphometry and density, soft tissue thickness (STT), and bone surface slope, and classified the MSB. Heat maps were created to identify potential implant locations.

**Results:**

Median MSB height was 50.7 [95% confidence interval 49.4–53.4] mm, and mean thickness ranged from 8.7 [8.3–9.2] to 13.1 [12.7–13.6] mm. Mean width ranged from 53.2 [51.1–55.4] to 57.1 [55.3–59.1] mm. Significantly greater values of thickness and width were found in males. Body height had a significant positive effect on thickness and width measured at the thickest level. Median STT was 14.4 [12.7–17.4] mm, on which BMI had a significant positive effect. The trapezoid was the most common shape. Mean densities were 325 and 60 HU for cortical and cancellous bone, respectively, and cancellous density was significantly higher in males. As such, the MSB is most similar to the posterior maxilla with regard to implantation.

**Conclusion:**

In theory, the MSB is able to support a combination of three to four 6-mm implants, or of one 6-mm to 8-mm and two 10-mm implants, placed in a linear, triangular or diamond-shaped arrangement.

**Relevance statement:**

This study addresses knowledge gaps regarding the possibility of fixation methods of hands-free speaking valves offering greater stability than conventional methods. Radiological assessment of CT scans of the *manubrium sterni* *bone* demonstrates its potential as an implantation site, although a thorough preimplantation workup of individual anatomy is imperative.

**Key Points:**

- This is the first study that analyzes the *manubrium sterni* *bone* three-dimensionally and assesses its potential as a bone implantation site.
- We found that it has low cortical and cancellous bone density, which makes it most similar to the posterior maxilla.
- *The Manubrium sterni bone* is suitable for implantation, although a preimplantation assessment of individual anatomy is imperative.

**Graphical Abstract:**

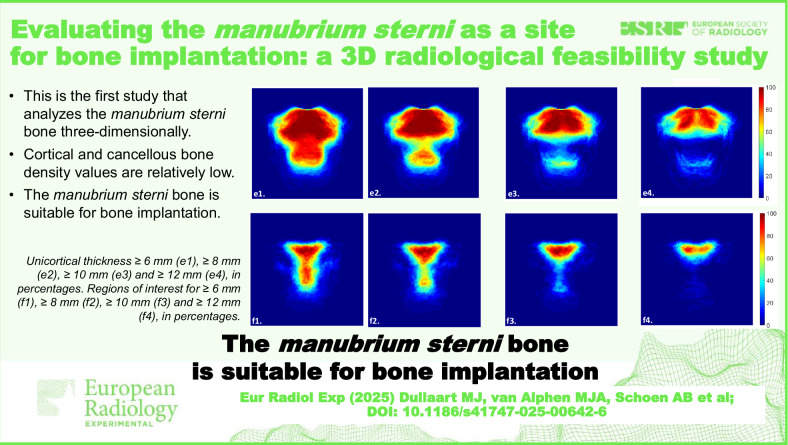

## Background

After total laryngectomy, the optimal end-goal of vocal rehabilitation is achieving “hands-free” speech through the use of an automatic speaking valve. This valve requires secure fixation to the peristomal region to ensure stability during speech. Although a variety of fixation methods exist, in daily practice, only 20% of laryngectomized patients are able to speak hands-free [1–8]. Commonly, an adhesive baseplate is used to retain the automatic speaking valve. The high backpressure needed for tracheoesophageal vocalization is the root cause for most issues related to fixation [9, 10], as it leads to bulging of the adhesive baseplate, and ultimately air leakage [11, 12]. Irregular tracheostoma anatomy and skin irritation are additional factors contributing to a short adhesive baseplate lifetime and failure to achieve adequate hands-free speech [13]. Patients counteract this phenomenon by applying manual pressure, which emphasizes the patient’s disability and necessitates the use of one hand during speech, thereby reducing their quality of life [14].

Several studies have sought to improve baseplate support through the development of devices such as neck braces and improved adhesives [15–17]. As this had not led to increased use of automatic speaking valves, we aim to explore experimental methods offering greater stability than conventional fixation devices. We hypothesized that future prosthetic devices could be anchored to implants placed in the manubrium sterni bone (MSB), improving fixation and enhancing hands-free speech for laryngectomized patients.

The MSB was selected as the implantation site because of its proximity, stability and immobility with regard to the tracheostoma. As a first step in this approach, the radiological assessment of the viability of the MSB for bone implantation is necessary. Although several studies have investigated MSB morphometry two-dimensionally [18–20], until now, none have used three-dimensional analysis to assess its potential as an implantation site. Therefore, we aim to determine MSB morphometry, density, and soft tissue thickness (STT), to classify the bone according to the Lekholm & Zarb (L&Z) and Norton & Gamble (N&G) classifications [21, 22], and to identify potential implant locations.

## Materials and methods

### Study design

This is a retrospective cross-sectional study, reported following the Strengthening the Reporting of Observational studies in Epidemiology guidelines for cross-sectional studies [23]. It was conducted at the Netherlands Cancer Institute in Amsterdam, the Netherlands, and approved by the Institutional Review Board (d22-245). Fifty computed tomography (CT)-scans made during the years 2014–2022 were included, from an equal number of male and female patients. Inclusion criteria were: (i) age around 65 years, the average age of a patient undergoing a total laryngectomy [24, 25]; and (ii) diagnosis of a primary or secondary head and neck malignancy. Exclusion criteria were: (i) history of sternal trauma, biopsy, or sternotomy; (ii) congenital or acquired bone disease; (iii) treatment with intravenous bisphosphonates; and (iv) prior radiotherapy of the neck or thoracic area for malignancies other than locally advanced laryngeal or pharyngeal carcinoma or associated neck metastasis. The CT scans were selected by and retrieved through the in-hospital Data Desk on the 11th of November 2022.

### Variables and data measurement

Data regarding sex, age, systemic treatment, osteoporosis, diabetes mellitus, smoking status, body mass index (BMI) and body height were collected from the patient files. For smoking status, the Mayo Clinic definitions were used [26]. The World Health Organization classification was used for osteoporosis staging [27].

**Fig. 1:**
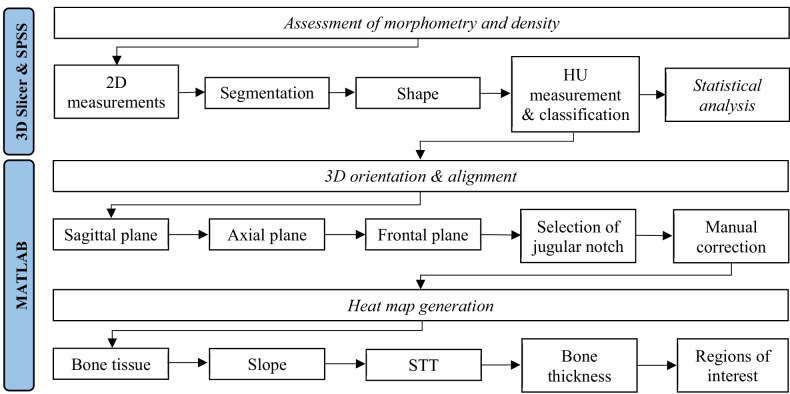
Flowchart showing the data analysis process. 2D, Two-dimensional; 3D, Three-dimensional; STT, Soft tissue thickness

Reconstruction kernel, use of intravenous contrast, tube current and peak voltage were noted for each scan. Scans were extracted as Digital Imaging and Communications in Medicine−DICOM files. Figure 1 shows an overview of the data analysis process.

**Fig. 2:**
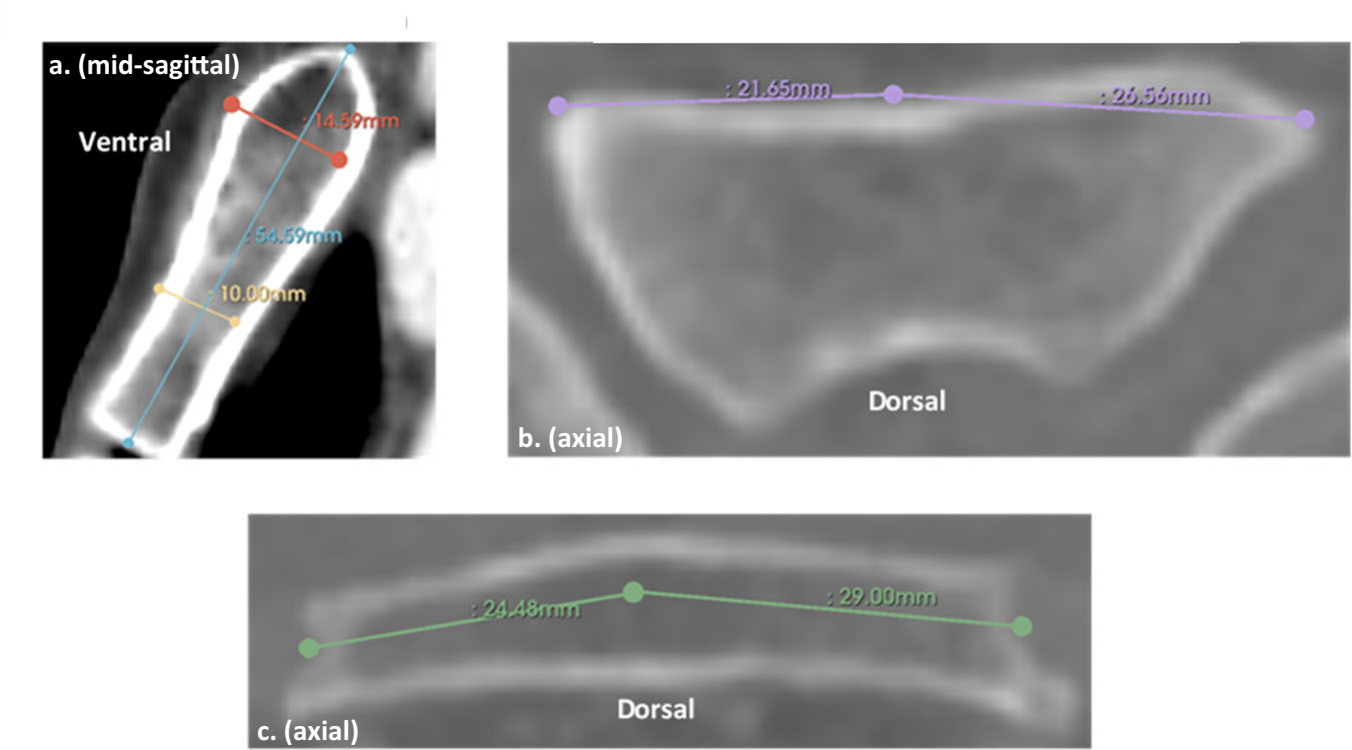
Distance measurements of the *manubrium sterni*. Height in blue, thickness at the thickest level in red and at the thinnest level in yellow (**a**). Width at the thickest level in purple (**b**), and at the thinnest level in green (**c**)

Two-dimensional morphometric measurements were manually performed by M.D. in 3D Slicer version 5.0.3 [28] using digital calipers, as shown in Fig. 2. Bone thickness and width were measured at the thickest and thinnest levels. Thickness was measured unicortically, including only the anterior cortex and cancellous bone. Width was measured using two separate lines running parallel to the anterior cortex, most representing MSB shape. Cortical and cancellous bone density, represented by median HU values, were classified according to the L&Z and N&G classifications, details of which can be found in Table 1.

**Table 1:** Bone type classifications according to Lekholm & Zarb and Norton & Gamble

Bone type	Description or criterion
*Lekholm & Zarb*	
1	Homogeneous cortical bone
2	Thick cortical bone with a marrow cavity
3	Thin cortical bone with dense trabecular bone of good strength
4	Very thin cortical bone with low-density trabecular bone of poor strength
*Norton & Gamble*	
1	> 850 HU
2−3	500–850 HU
4	0–500 HU
4^a^	< 0 HU

^a^ Category 4 is described in the original article as “the failure zone”: values below 0 HU indicate considerable surface contact with fat marrow, and the implant’s prognosis must be regarded as poor

Two authors, M.D. and A.S., each segmented the MSB and the overlying soft tissue using 3D Slicer, each accounting for half of the scans. Region of interest size and placement, as well as the technique used for measuring bone density, are clarified in the appendix. Although not each segmentation was separately checked, any anatomical anomalies leading to difficulties with the segmentation were discussed with a radiologist (L.B.).

**Fig. 3:**
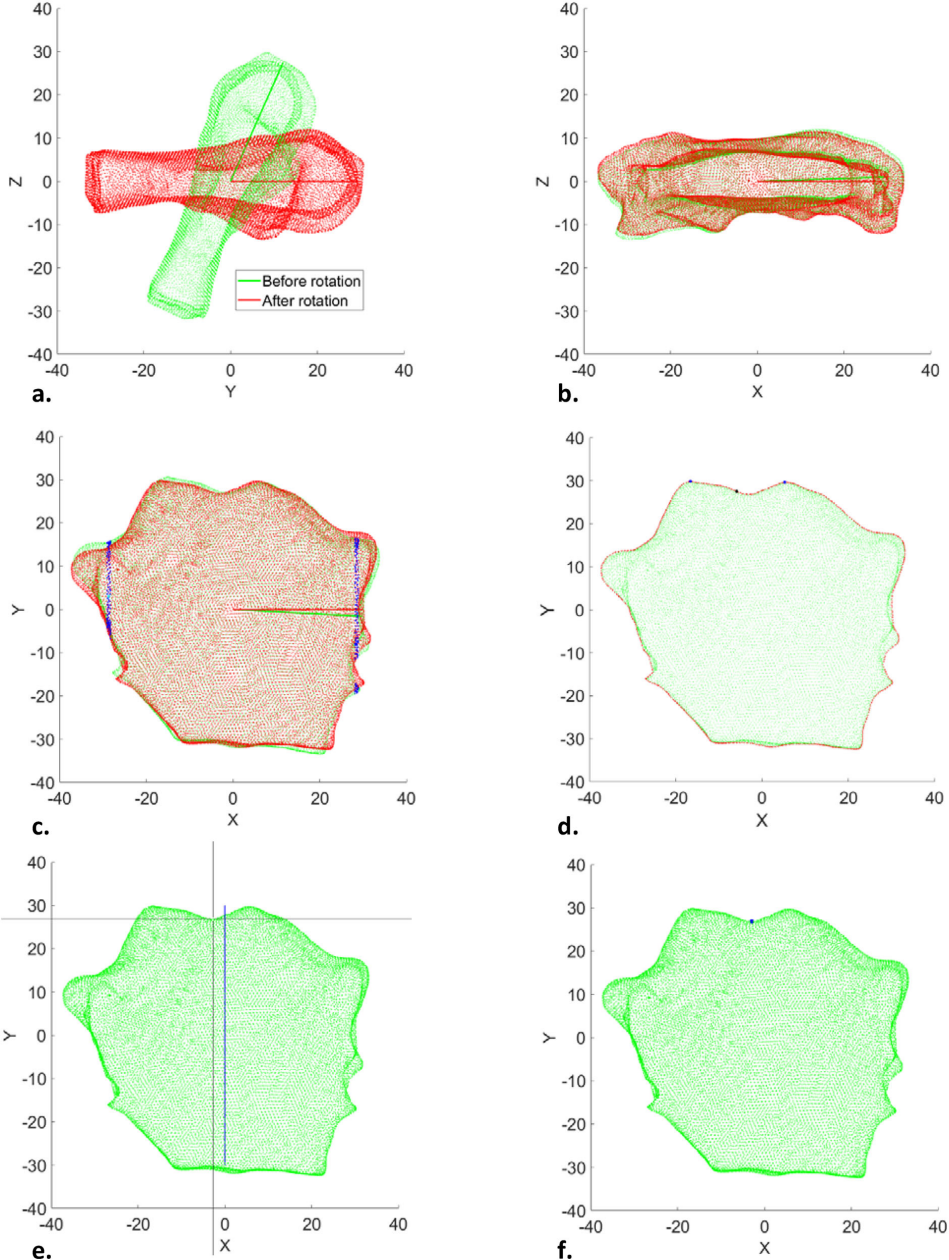
Orientation through rotation around the mediolateral axis (**a**), longitudinal axis (**b**), and anterior-posterior axis (**c**, mediolateral coordinates in blue); alignment by automatic (**d**) and manual (**e**) selection of the jugular notch with a cross-hair and mid-sagittal line as reference (in blue), and subsequent visual check (**f**)

Subsequently, M.D. classified the bone shape as either trapezoid, quadrangular or triangular. Unicortical bone thickness and STT, as well as slope, were mapped three-dimensionally, and the average STT for each patient was calculated. Slope was assessed because angles (between implant and bone surface) smaller than fifteen degrees can remain uncorrected, whereas angles of more than fifteen and up to 45 degrees do require correction (*e.g*., by using abutments or through bone milling).To create the maps, the segmented data were exported as STL files, and the data point clouds were rotated semiautomatically, around three axes, using MATLAB, version 2023a (The MathWorks Inc., Natick, USA). For the first two rotations, principal component analysis was used to determine fit lines. Figure 3a, b display the cortical point clouds before and after the first two rotations. Because the data point clouds seldom extended in one direction or another in the frontal plane, principal component analysis was deemed unsuitable for rotation in that plane.

Therefore, data points along the anterior-posterior axis were all sorted by their mediolateral axis (*x*) coordinates between the 5th and 6th percentiles to eliminate the costochondral junctions bilaterally. A fit line perpendicular to these data points was drawn and used for rotation (Fig. 3c). After orientation, the MSBs were aligned in the frontal plane on the jugular notch, which was found by marking the marginal data points representing the boundary of the data point cloud. The two marginal data points with the greatest value on the anterior-posterior axis (*y*), both below and above the zero coordinate on the mediolateral axis (*x*), were selected. The data point halfway between these two was determined to be the jugular notch (Fig. 3d). In some cases, however, manual correction was necessary, using cross-hairs and a mid-sagittal line as reference (Fig. 3e). Any manual correction was visually checked (Fig. 3f).

To determine bone thickness and STT, the perpendicular distance between two faces was calculated: from the anterior margin of the anterior cortex to the anterior margin of the posterior cortex, and from the anterior margin of the skin to the anterior margin of the anterior cortex, respectively. Subsequently, heat maps were generated, displaying the presence of bone tissue, STT, the average slope, and the regions with sufficient bone thickness for various implant lengths, as well as regions with a slope of fifteen degrees or less. In addition, heat maps combining the latter two regions were created. Each map represents the data of all patients, and was generated with a resolution of one by one mm.

### Bias and sample size

Selection bias was minimized through random and blinded selection of patients. Measurement bias was minimized by registering scanning parameters and by having two authors segment the scans. For practical reasons, the measurements were done by one author. As this is a feasibility study, a sample size calculation was not performed, and fifty samples were included to obtain generalizable results.

### Statistical methods

Statistical analyses were performed using SPSS software version 30 (IBM Corp, Armonk, USA). Syntaxes can be found in the appendix. Missing values were imputed using the mode and the mean for categorical and quantitative variables, respectively. A bootstrap procedure with 1,000 iterations was used to calculate 95% confidence intervals (CI) for the mean and the median values. Normality was checked by visual inspection of histograms. Main outcome data (*i.e.*, morphometry, bone density, and STT) were presented by either the mean [95% CI] for normally distributed data, or by the median [95% CI] in the case of non-normally distributed data.

For secondary variables (*i.e.*, age, BMI, and body height), mean, standard deviation (SD) and range, or median and range were reported for normally and non-normally distributed data, respectively. Details of the variables used can be found in Supplementary Table S1. A directed acyclic graph, which can be found in the appendix, was created to determine confounding variables and biasing paths that needed adjusting [29]. Multiple linear regression analysis was performed to relate gender and body height to bone height, thickness and width, bone density to gender, smoking status and age, and STT to BMI. Assumptions for linearity were checked and met, and confounding variables were adjusted for. Values of *p* below 0.05 were considered statistically significant.

Furthermore, to determine the reliability of the segmentation method and assess where potential inconsistencies could be found, two authors (M.D. and A.S.) segmented the scan with the most common configuration within our sample five times. Then, the cortical and cancellous tissue segmentations were compared using 3D Slicer, and Dice similarity coefficients, reported as percentages, with values of at least 70% indicating good overlap, and modified Hausdorff distances (HD), with average values, reported as median and range, were computed.

## Results

### Participants

Table 2 shows the aggregated individual patient characteristics. Of the fifty scans, one was retrospectively excluded after consultation with our radiologist, because of unexpected deformation to the bone that was not registered in the patient file. Thus, scans of 25 males and 24 females, aged 65.7 ± 7.0 years (mean ± standard deviation) were used for analysis. One patient was osteopenic, and three had type 2 diabetes. Most patients were former smokers. Body height was 172.5 ± 8.1 cm (mean ± standard deviation), and median BMI was 22.3, ranging from 15.3 to 45.5.

**Table 2:** Patient characteristics

Characteristic	Total number of patients or mean with standard deviation and range
Sex	M (25), F (24)
Age in years	65.7 ± 7.0, 54–88
Systemic treatments	IT (8), CT (12), HT (1), CT & IT (7), CT & HT (2)
Osteoporosis	No (48), Osteopenic (1)
Diabetes mellitus	No (46), II (3)
Smoking status^a^	Current (17), Past (22), Never (10)
BMI^b^	23.7 ± 5.8, 15.3–45.5
Body height in cm	172.5 ± 8.1, 156–190

*CT* Chemotherapy, *HT* Hormone therapy, *IT* Immunotherapy

^a^ This is seldom updated after the first contact, so the number of current smokers is possibly inflated

^b^ Only values within two months of the scanning date were noted

### Bone geometry

Details regarding bone geometry can be found in Table 3. Details of the outcomes of the statistical analyses can be seen in Supplementary Table S2. Median bone height was 50.7 [95% CI 49.4–53.4] mm. Mean thickness and width measured at the thickest level were 13.1 [12.7–13.6] and 53.2 [51.1–55.4] mm, respectively. At the thinnest level, mean thickness and width were 8.7 [8.3–9.2] and 57.1 [55.3–59.1] mm, respectively. Males had significantly greater thickness measured at both the thickest (*β* = 1.387 [0.524–2.250]; *p* = 0.002) and the thinnest levels (*β* = 1.914 [1.173–2.654]; *p* < 0.001), and greater width measured at the thinnest level (β = 7.098 [3.866–10.329]; *p* < 0.001). In addition, body height had a significant positive effect on thickness (*β* = 0.072 [0.003–0.141]; *p* = 0.041) and width (*β* = 0.422 [0.061–0.782]; *p* = 0.023) measured at the thickest level whilst adjusting for sex. Moreover, median STT was 14.4 [12.7–17.4] mm, and BMI had a significant positive effect on STT (*β* = 1.069 [0.740–1.399]; *p* < 0.001). Furthermore, bone shape was mostly classified as trapezoid (55%; *n* = 27), followed by quadrangular (39%; *n* = 19) and triangular (6%; *n* = 3). The different shapes are shown in Fig. S1.

**Table 3:** MSB geometry and average soft tissue thickness (in mm, with mean, standard deviation and range below)

ID number	Height	Thickness SL	Thickness IL	Width SL	Width IL	Shape	Soft tissue thickness
1	43.0	12.0	6.5	53.5	50.5	Trapezoid	12.8
2	57.6	13.2	9.6	48.2	53.5	Trapezoid	10.7
3	46.6	12.3	9.5	50.9	51.8	Trapezoid	20.0
4	53.8	12.8	9.2	55.4	60.5	Trapezoid	17.6
5	46.8	11.5	7.5	54.8	53.7	Trapezoid	14.4
6	45.6	13.6	8.4	54.8	44.5	Trapezoid	17.7
7	47.2	14.0	11.3	52.7	59.2	Trapezoid	33.0
8	53.8	15.4	9.4	54.0	56.4	Triangular	17.4
9	50.6	12.2	7.7	47.2	58.1	Trapezoid	13.5
10	46.1	12.2	7.9	48.9	63.4	Trapezoid	20.0
11	45.5	13.3	9.5	49.1	55.9	Trapezoid	40.8
12	59.5	14.2	11.2	51.1	68.5	Quadrangular	11.7
13	56.2	12.0	7.3	60.9	52.8	Triangular	12.9
14	59.4	15.3	11.5	49.7	59.2	Trapezoid	47.3
15	47.6	11.4	7.6	45.4	50.2	Quadrangular	22.3
16	58.3	12.6	9.0	50.4	58.8	Trapezoid	9.4
17	57.1	13.9	9.6	40.4	65.5	Quadrangular	23.1
18	45.5	13.6	10.2	59.7	61.0	Trapezoid	8.6
19	54.6	14.6	10.1	50.0	59.8	Trapezoid	7.8
20	49.5	12.9	9.5	70.1	68.8	Trapezoid	14.9
21	58.5	17.1	13.3	74.5	71.5	Quadrangular	13.9
22	49.9	13.4	8.4	50.7	59.4	Trapezoid	38.2
23	46.8	13.2	9.4	56.4	72.1	Triangular	11.1
24	47.0	13.1	7.3	50.5	58.8	Quadrangular	15.0
25	50.9	15.5	10.6	51.8	57.4	Quadrangular	14.3
26	50.7	10.6	6.6	45.1	56.3	Quadrangular	7.4
27	63.4	12.7	8.2	54.6	61.6	Quadrangular	19.5
28	51.7	10.9	7.3	49.5	45.1	Quadrangular	29.0
29	47.1	11.4	7.2	49.6	58.9	Quadrangular	10.3
30	62.9	15.3	6.4	61.9	58.5	Quadrangular	22.0
31	53.4	12.0	8.7	46.4	66.7	Trapezoid	27.4
32	50.4	13.5	11.3	62.1	55.2	Triangular	23.5
33	49.3	12.5	7.3	45.8	57.3	Quadrangular	12.7
34	56.5	13.8	7.8	70.1	61.0	Trapezoid	22.4
35	58.3	12.4	9.4	46.8	57.4	Quadrangular	18.7
36	54.4	14.8	10.5	63.1	61.7	Quadrangular	4.4
37	57.5	14.0	9.3	53.1	53.1	Trapezoid	21.0
38	50.4	13.5	11.0	58.5	57.1	Quadrangular	7.4
39	69.1	15.4	10.0	61.6	51.7	Quadrangular	27.7
40	52.0	15.3	9.0	64.9	60.1	Trapezoid	7.9
41	53.0	12.2	7.7	45.9	44.1	Quadrangular	15.3
42	49.4	10.4	6.7	66.5	54.0	Trapezoid	8.6
43	48.2	12.5	6.9	56.3	48.7	Trapezoid	19.2
44	41.6	11.0	7.3	44.1	46.2	Trapezoid	10.7
45	56.5	17.2	8.4	37.6	61.3	Trapezoid	15.7
46	48.2	9.7	7.6	36.8	49.4	Quadrangular	6.8
47	48.0	13.2	8.4	53.8	53.1	Quadrangular	16.4
48	47.8	12.7	7.4	56.9	59.7	Trapezoid	9.8
49	51.0	11.0	6.1	44.5	47.6	Trapezoid	12.4
Mean ± SD Range	52.0 ± 5.7 41.6–63.4	13.1 ± 1.6 9.7–17.2	8.7 ± 1.6 6.1–13.3	53.2 ± 8.1 36.8–74.5	57.1 ± 6.6 44.1–72.1	−	15.0 4.4–47.3

*IL* Inferior (thinnest) level, *SL* Superior (thickest) level, *SD* Standard deviation

### Bone density

Details regarding the various scanning configurations can be found in Table 4. Mean densities were 325 [305–345] and 60 [51–70] HU for cortical and cancellous bone, respectively. Males had a significantly higher cancellous bone density (*β *= 22.017 [2.820–41.213]; *p* = 0.025). According to the L&Z classification, 48 cases were classified as type four, and one case was classified as L&Z type three. Forty-seven cases were classified as type four according to the N&G classification, and two cases were of the additional type four.

**Table 4:** Scan settings, bone density values (with range below) and classification

ID number	Kernel	CE	Tube current (mA)	Voltage (kVp)	Median HU value		N&G class	L&Z class
					Cortical bone	Cancellous bone		
1	Soft tissue	-	178–635	80	458	45	4	4
2	Soft tissue	-	91–414	80	424	54	4	4
3	Soft tissue	-	18–82	100	307	24	4	4
4	Soft tissue	-	150–210	120	301	-2	4	4^a^
5	Soft tissue	-	123–214	100	381	74	4	4
6	Soft tissue	-	15–215	120	250	38	4	4
7	Soft tissue	+	134–686	100	383	46	4	4
8	Soft tissue	+	61–256	80	326	99	4	4
9	Soft tissue	+	150 (fixed)	120	215	18	4	4
10	Soft tissue	-	134–642	120	420	91	4	4
11	Soft tissue	+	196–439	120	290	93	3	4
12	Soft tissue	+	150 (fixed)	120	250	43	4	4
13	Soft tissue	+	48–200	80	376	68	4	4
14	Soft tissue	+	145–644	90	378	45	4	4
15	Soft tissue	+	151–220	100	287	49	4	4
16	Soft tissue	-	25–145	90	330	28	4	4
17	Soft tissue	+	180–516	80	474	123	4	4
18	Soft tissue	-	108–633	100	337	118	4	4
19	Soft tissue	+	50–115	120	264	42	4	4
20	Soft tissue	+	150–155	120	250	59	4	4
21	Soft tissue	+	86–745	80	342	58	4	4
22	Soft tissue	-	162–659	120	305	74	4	4
23	Soft tissue	+	74–327	80	414	60	4	4
24	Soft tissue	+	150 (fixed)	120	239	60	4	4
25	Lung tissue	+	61–120	120	347	51	4	4
26	Soft tissue	+	142–417	80	389	27	4	4
27	Soft tissue	+	150–402	120	287	78	4	4
28	Soft tissue	-	95–445	100	308	31	4	4
29	Soft tissue	+	150–160	120	292	93	4	4
30	Soft tissue	-	47–379	120	257	55	4	4
31	Lung tissue	+	153–380	120	354	77	4	4
32	Bone tissue	-	155–356	120	390	48	4	4
33	Soft tissue	+	150 (fixed)	120	387	134	4	4
34	Soft tissue	-	124–591	100	424	120	4	4
35	Soft tissue	-	49–250	90	451	76	4	4
36	Soft tissue	+	150–180	120	237	16	4	4
37	Lung tissue	+	50–227	80	447	64	4	4
38	Soft tissue	+	120–341	120	270	43	4	4
39	Soft tissue	+	150–361	120	311	41	4	4
40	Soft tissue	-	101–359	120	318	113	4	4
41	Soft tissue	+	70–172	120	258	34	4	4
42	Soft tissue	+	151–381	100	230	-12	4	4^a^
43	Soft tissue	+	63–741	90	224	21	4	4
44	Soft tissue	-	194–396	100	347	135	4	4
45	Soft tissue	-	150–210	120	217	29	4	4
46	Soft tissue	+	150 (fixed)	120	329	77	4	4
47	Soft tissue	+	150–175	120	183	10	4	4
48	Soft tissue	-	121–231	100	363	61	4	4
49	Soft tissue	+	50–129	120	294	102	4	4
Mean ± SD Range	−	−	−	−	325.0 ± 72.8 183–474	60.0 ± 34.9 -12–135	−	−

Most scans were performed with a Toshiba Aquillion CX or a Siemens SOMATOM Force scanner. Four scans were performed in another center with an unknown scanner. Each scan was performed with the patient in supine position. Most were contrast-enhanced (*n* = 30), and reconstructed using a soft tissue kernel (*n* = 23), followed by lung tissue kernel (*n* = 3) and bone tissue kernel (*n* = 1). The tube current ranged from 15 to 745 mA and was fixed at 150 mA in five cases, to prevent excessive radiation levels. Peak voltages measured 120 (*n* = 26), 100 (*n* = 10), 80 (*n* = 9) or 90 (*n* = 3) kV

*CE* Contrast-enhanced, *L&Z* Lekholm & Zarb classification, *N&G* Norton & Gamble classification, *SD* Standard deviation

^a^ Of the “additional bone type 4”

### Observer reliability

Concerning the reliability of the segmentation method, Dice similarity coefficient values ranged from 91 to 99%, and HD values were 0.33 [0.07–0.67] and 0.22 [0.07–0.51] mm for the cortical and cancellous layers, respectively. Figure S1 displays a heat map of the HD values.

### Bone thickness and slope

**Fig. 4:**
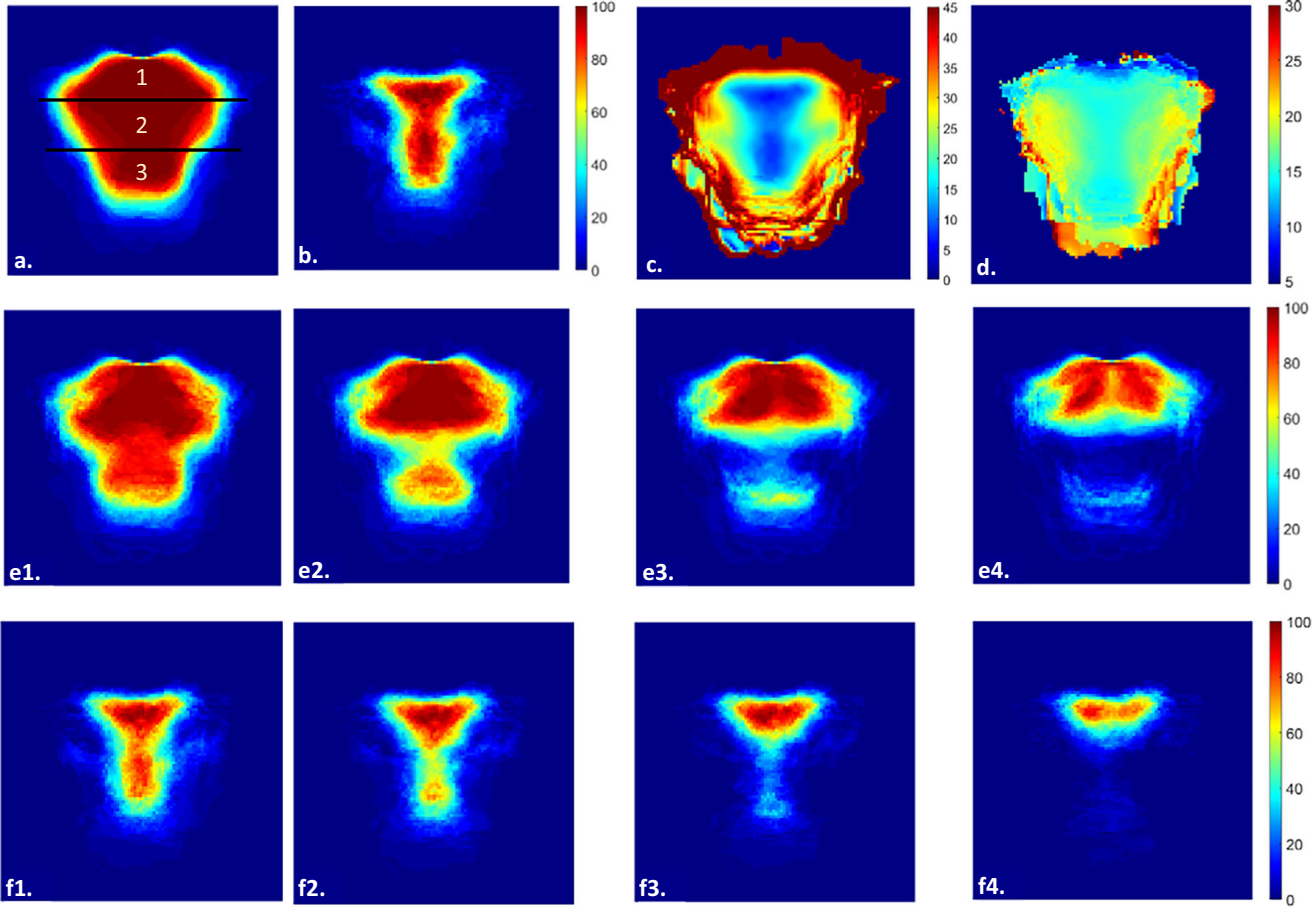
Top row: presence of bone tissue, in percentages (**a**), slope < 15 degrees, in percentages (**b**), average slope, in degrees (**c**), average soft tissue thickness, in mm (**d**). Middle row: unicortical thickness ≥ 6 mm (**e1**), ≥ 8 mm (**e2**), ≥ 10 mm (**e3**). and ≥ 12 mm (**e4**), in percentages. Bottom row: region of interest maps for 6 (**f1**), 8 (**f2**), 10 (**f3**), and 12 (**f4**) mm implants, in percentages

Figure 4a demonstrates that the MSB has a tapered contour, divisible into an apex, mid-section, and base. Figure 4b, c shows that its slope rarely exceeds thirty degrees in the center, mostly ranging between five and twenty degrees. Figure 4d displays the STT. Figure 4e reveals that the thickest regions are located around two apical protuberances in the paramedian line. Lastly, Fig. 4f shows where the slope is less than fifteen degrees for each of the four implant lengths, and reveals that in at least 75% of the cases within our sample, the apex is able to accommodate implants of various lengths, and the apex, mid-section and all three sections are implantable with 6-mm implants. When comparing maps f4 and e4, it can be noted that while the thickest regions are well-suited concerning thickness, their steep slope may pose difficulties. Roughly ten mm above these regions bilaterally, slope and thickness do seem suitable for at least one or more 10-mm implants. These are the regions with the greatest potential for implantation.

## Discussion

The main findings of this study can be summarized as three key points. First, considerable inter-individual MSB variability in morphometry was observed. Males had significantly greater bone thickness and width compared to females, and body height had a significant positive effect on thickness and width measured at the thickest level. Median STT was 14.4 mm, and BMI had a significant positive effect on STT. Second, mean densities were 325 and 60 HU for cortical and cancellous bone, respectively, and males had significantly higher cortical bone density than females. All but three cases could be classified as type four bone according to both the L&Z and N&G classifications. Third, we found that the MSB has two protuberances located apically in the paramedian line, where it is thickest and steepest. Generally, its slope rarely exceeds thirty degrees, mostly ranging between five to twenty degrees. The apex, mid-section and base are implantable with 6-mm implants, and roughly ten mm above the protuberances on both sides, slope, and thickness do seem suitable for at least one or more 10-mm implants.

With regard to geometric properties, our findings corresponded to similar studies. Xiu et al [19] measured height and thickness at the thickest and thinnest levels, and reported slightly smaller values. However, they measured the cancellous bone only. Selthofer et al [18] assessed MSB thickness at the same levels, including both cortices and the cancellous bone, and they also examined bone shape. They reported very similar values with regard to thickness, and our findings with regard to shape were comparable as well. Lastly, Bolatli et al [20] reported a mean bone height of 47.0 mm in a group of 142 males and 127 females aged sixty years and older. All three studies stated that males had a significantly larger MSB than females. Although we measured bone height and thickness using similar methods, this is the first study to investigate and perform three-dimensional analysis. The analysis also involved manual segmentation [30] and automated thickness and slope mapping, thereby providing deeper insight into the morphometric properties of the MSB.

With regard to bone density and its place within the L&Z and N&G classifications, our findings indicate that the MSB generally has thin cortices and low cancellous bone density, most similar to that of the posterior maxilla [31]. This implies that the implantation protocols used for the implantation of that area might serve as groundwork for the implantation of the MSB. Our findings concerning bone thickness and slope have provided us with crucial insight into the possibilities with regard to implant length and arrangement. Depending on the expected forces on the implants, a combination of three to four 6-mm implants, or of one 6-mm to 8-mm and two 10-mm implants, appears to be the best available option, placed in a linear, triangular or diamond-shape arrangement.

However, the following points should be considered. First, if the use of 12-mm and larger implants is preferable, the apical and basal thickness will have to be measured to determine if they are sufficient. Second, even though our sample was sufficiently large to provide generalizable results, there were two cases with bone density values unsuitable for implantation (*i.e.*, of the additional type four according to N&G). Third, our sample included one MSB with a unicortical bone thickness of less than six mm, and was therefore unable to accommodate even the shortest implants. Furthermore, although the posterior cortex could theoretically serve as a tangible sign to stop drilling, bicortical implantation is not a viable option due to the fragility of the anatomical structures situated directly behind the posterior cortex. Finally, to ensure an adequate implant-abutment ratio and that peri-implant hygiene can be maintained, a maximum STT of approximately five mm should be present around the implants [32].

Implant and soft tissue reduction will be done following surgical protocols for mastoid implantation, entailing a two-stage procedure, with soft tissue modification performed four months after implant placement [33]. In our experience, this involves minimal surgical risk (*e.g.*, wound healing issues, implant failure), although in some cases a third of fourth operation might be required. Since the median STT in this study was 14.4 mm, soft tissue correction will be necessary for most patients, before which its esthetic consequences must be discussed. To be able to do so, a precise and patient-specific assessment of STT is required.

The abovementioned reasons highlight the necessity of a thorough preimplantation workup, including the use of a scanning protocol in which at least body position, reconstruction kernel, tube current and voltage are standardized. A proposal for a preimplantation scanning protocol, which was based on available in-hospital CT head and implant protocols, and was underwritten by our radiologist, can be found in the Appendix. A preimplantation workup should also involve segmentation and virtual placement of the desired implants inside the segmented model, similar to the clinical workup prior to auricular and nasal implantation. Bone density can also be determined after segmentation. In addition, the presence of any anatomical anomalies such as suprasternal tubercles or manubrial bands [34, 35], or anatomical rotation of the MSB in the coronal and frontal planes can be detected, as rotation in the latter may have consequences for how the prosthesis will be situated relative to the tracheostoma.

There are some limitations to our study. First, the directed acyclic graph identified nutritional status as a confounder, but due to a lack of information on this variable, we were unable to adjust for it. However, BMI is often used as a proxy measure for nutritional status, and a diagnosis of osteoporosis is an indication of long-term poor nutritional status. As both these variables were included and adjusted for in the analysis, any potential confounding has been at least partially addressed. Nonetheless, we emphasize the importance of individualized surgical planning for clinical application. Second, though one can expect a certain learning curve in segmenting CT scans, this procedure was not performed by a radiologist or otherwise trained professional. However, the high Dice similarity coefficient values showed excellent segmentation method reliability, and the HD values that were calculated were low and could furthermore be partly attributed to voxel size. Moreover, it is an overestimation due to outliers located at the costochondral junctions, where accurate segmentation was more difficult. Considering this, the HD values do not have any clinical consequences. Third, the last rotation and the alignment method both required visual checks afterwards, resulting in manual corrections in some cases. In addition, some rotations might have been unnecessary, as the procedure does not distinguish between anatomical variation (*e*.*g*., thoracic rotation due to spinal deformities) and body position (*e.g.*, natural rotation in the shoulders or back). Fourth, although the L&Z and N&G classifications were developed for, and are primarily used in, dental implantology, they entail visual inspection of CT-images and measurement of HU-values, respectively, which makes them highly applicable to other potential implantation sites. Moreover, we aim to use dental implantation protocols as groundwork for the implantation of the MSB. Lastly, as it is known that attenuation values are affected by voltage, reconstruction kernel and the use of contrast, and the scans in our sample were performed using diverse protocols, the resulting bone density values may not be altogether comparable [36–39]. However, the cortices of the *manubriae* included in our sample measured 325 HU on average, and the surrounding tissue had a much lower HU value. Therefore, the variation in HU-values will have had little effect on the segmentation process.

Furthermore, because there was little difference between the reconstruction kernels that were used, and contrast enhancement rarely affects bone tissue, only the variation in voltage might have affected attenuation values. As lower voltages (80–100 kVp) correspond to decreased HU-values, which in turn correspond to higher N&G classification, low voltage might have led to an overvaluation with regard to N&G category, whereas higher voltages (> 100 kVp) might have led to undervaluation. Standardization of a scanning protocol would entail the use of higher voltages, and most scans in this study were performed under high voltage. Hence, it is safe to assume that use of a standardized protocol would not affect the N&G classification much—except for the lower voltage cases, which would then probably be assigned to a higher N&G category.

In conclusion, our study shows that the MSB bone demonstrates potential as an implantation site, although a thorough preimplantation workup to assess individual anatomy is imperative. This includes segmentation of scans performed using a standardized protocol and making a patient-specific assessment of their morphometry and density. Future studies will assess biomechanical variables such as strain, implant displacement and stress when loading the implants through finite element analysis. This involves knowledge of cortical and cancellous bone quality [40], and could be used to assess the force loaded on the bone during speech, and the subsequent transfer of force onto the implants. To develop a reliable implantation protocol using this technique, future studies are needed, in which the microarchitecture of the MSB is investigated, and a full preimplantation workup and bone implantation are evaluated. Eventually, a clinical trial involving human participants should be performed, in which patient selection is done according to criteria similar to those used in this study. Candidates for implantation would include patients unable to achieve hands-free speech due to adhesive dislodgement or excessive pressure during voicing. Initially, implantation may be considered as a secondary procedure after laryngectomy. If proven successful, it could eventually be incorporated into the primary laryngectomy workflow.

## Additional file 1: Table S1. Variables used in the analysis. Table S2. Outcomes multiple linear regression. Fig. S1. Three-dimensional shapes of the manubrium (yellow = trapezoid, green = quadrangular, red = triangular), and a heat map displaying the Hausdorff distances, illustrating how outliers are mostly situated at the demarcation between the manubrium and first rib.

Additional file 1: Table S1. Variables used in the analysis. Table S2. Outcomes multiple linear regression. Fig. S1. Three-dimensional shapes of the manubrium (yellow = trapezoid, green = quadrangular, red = triangular), and a heat map displaying the Hausdorff distances, illustrating how outliers are mostly situated at the demarcation between the manubrium and first rib.

## Data Availability

Data such as the MATLAB code and statistical analysis syntax are available upon request.

## Abbreviations

BMI: Body mass index
CI: Confidence interval
CT: Computed tomography
HD: Hausdorff distance
L&Z: Lekholm & Zarb
MSB: Manubrium sterni bone
N&G: Norton & Gamble
STT: Soft tissue thickness
