# Supplementary material for: Evaluating the manubrium sterni as a site for bone implantation: a 3D radiological feasibility study
Source: Eur Radiol Exp. 2025 Dec 3;9:118. doi: 10.1186/s41747-025-00642-6 (PMC12675852; doi:10.1186/s41747-025-00642-6)
Supplement: Supplementary file 1 — Additional file 1: Table S1. Variables used in the analysis. Table S2. Outcomes multiple linear regression. Fig. S1. Three-dimensional shapes of the manubrium (yellow = trapezoid, green = quadrangular, red = triangular), and a heat map displaying the Hausdorff distances, illustrating how outliers are mostly situated at the demarcation between the manubrium and first rib. [file 41747_2025_642_MOESM1_ESM.pdf]

Evaluating the manubrium sterni as a site for bone implantation: a 3D radiological feasibility study

ELECTRONIC SUPPLEMENTARY MATERIAL

Table S1. Variables used in the analysis

Independent variables	*Dependent variables*
Sex*	Morphometry**
Body height	
Adjusted variables: sex	
Smoking status	Bone density
Adjusted variables: age & sex	
Age*	
Sex*	
BMI	Soft tissue thickness
Adjusted variables: none	

* no variables that need adjusting, ** MSB height, thickness and width

**Table S2.** Outcomes multiple linear regression						
Independent variables	*Beta*	*SB*	*p-value*	*Lower CI 95%*	*Upper CI 95%*	*Dependent variable*
**Morphometry**						
Male	3.087	0.273	0.058	-0.103	6.277	MSB Height
Body height[a]	0.119	0.131	0.172	-0.146	0.384	
Male	1.387	0.427	0.002	0.524	2.250	MSB Thickness (thickest level)
Body height[a]	0.072	0.362	0.041	0.003	0.141	
Male	1.914	0.604	< 0.001	1.173	2.654	MSB Thickness (thinnest level)
Body height[a]	0.035	0.180	0.256	-0.026	0.096	
Male	4.182	0.260	0.071	-0.380	8.743	MSB Width (thickest level)
Body height[a]	0.422	0.428	0.023	0.061	0.782	
Male	7.098	0.542	< 0.001	3.866	10.329	MSB Width (thinnest level)
Body height[a]	0.249	0.311	0.060	-0.011	0.509	
**Bone density**						
Age	-0.013	-0.001	0.993	-3.048	3.023	Cortical bone density
Male	40.188	0.279	0.052	-0.398	80.775	
Former smokers[b]	-12.272	-0.085	0.670	-69.920	45.376	
Current smokers[b]	2.546	0.017	0.936	-60.498	65.590	
Age	0.245	0.049	0.736	-1.208	1.698	Cancellous bone density
Male	22.017	0.319	0.025	2.820	41.213	
Former smokers[b]	-18.846	-0.272	0.157	-45.251	7.559	
Current smokers[b]	-22.898	-0.316	0.117	-51.774	5.979	
**Soft tissue thickness**						
BMI	1.069	0.690	< 0.001	0.740	1.399	Soft tissue thickness

SB standardized B; [a] adjusted for sex; [b] adjusted for age and sex

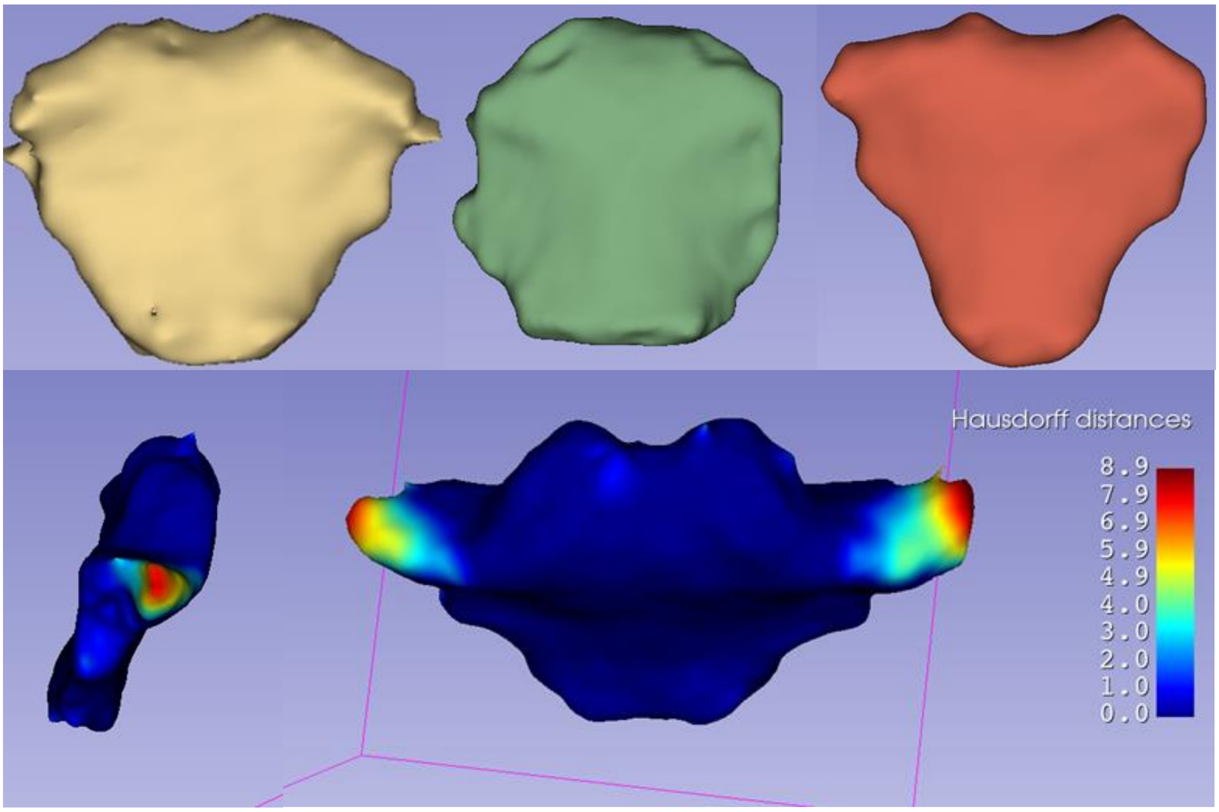

Fig. S1 Three-dimensional shapes of the manubrium (yellow = trapezoid, green = quadrangular, red = triangular), and a heat map displaying the Hausdorff distances, illustrating how outliers are mostly situated at the demarcation between the manubrium and first rib.

Appendix for *Evaluating the manubrium sterni as a site for bone implantation: a 3D radiological feasibility study.*

<u>Table of contents</u>

Segmentation method

MATLAB code

Manual corrections

Soft tissue thickness values and histogram

Directed acyclic graph

Full statistical syntax

Proposed scanning protocol

1. **Segmenting the cortices**

A. In segment editor, select ‘Allow overlap’ in the ‘Masking’ window using the dropdown menu next to ‘Modify other segments’.

B. Choose ‘Threshold’. Select a threshold Hounsfield units (HU) suitable for filtering only the bony cortices.

C. Use the features ‘Keep selected island’ and ‘Scissors’ to crop the manubrium sterni bone (MSB).

D. Use ‘Paint’ to draw connecting lines to manually close any remaining holes in the cortex.

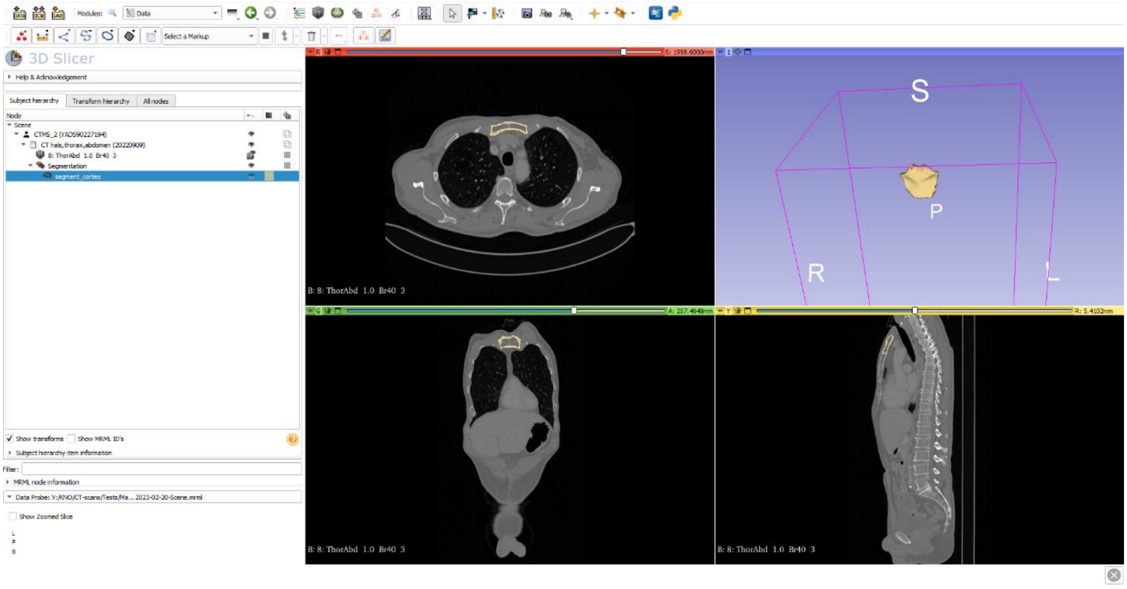

2. Segmenting the marrow

A. Clone the segmented cortex.

B. Use ‘Flood filling’ (part of the extension ‘SegmentEditorExtraEffects’) and Closing (fill holes) to segment the inner part of the bone. Another option is to manually fill the segmentation using the ‘Paint’.

C. Clone the segmentation which was just created.

D. Use ‘Logical operators’ in Segment Editor to subtract the cortex from the segmentation containing both cortex and marrow. Remove any voxels outside of the marrow manually and by using ‘Keep selected island’. If everything went well, you will now have 3 segments: one representing the cortex, another representing both cortex and marrow and a third containing only the marrow.

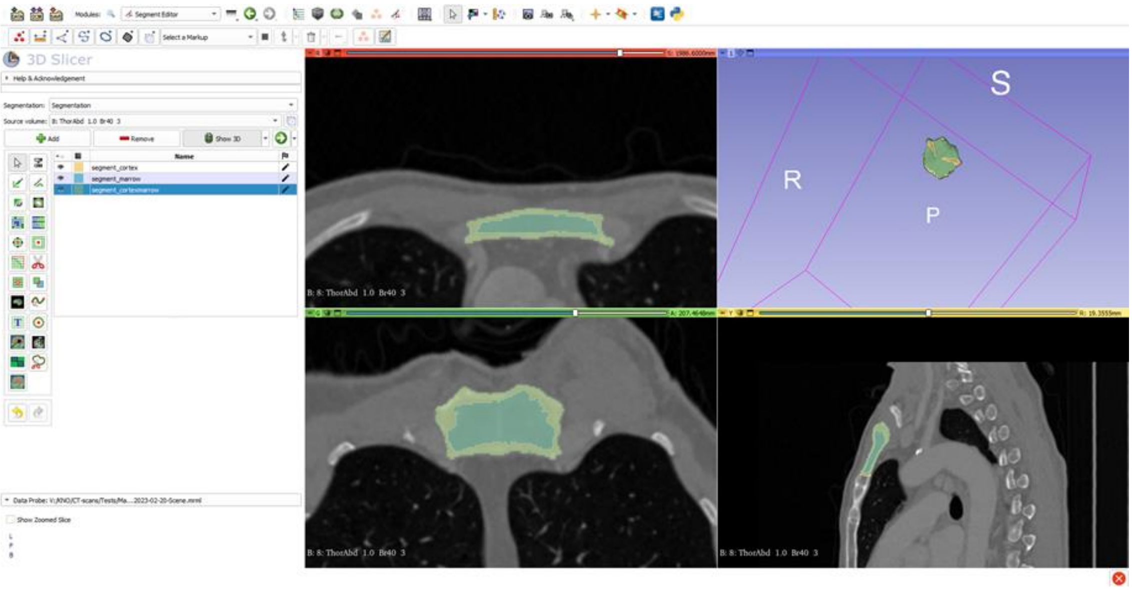

3. Segmenting the presternal skin

A. Create a fourth segment.

B. Use the feature 'Threshold' to filter out structures other than the skin using certain HU values (± -400 – 200).

C. Convert the segmentation to a model.

D. Isolate the area of interest using a ROI and the 'ROI cut' feature in Dynamic Modeler (untick the 'Cap surface' box). Transform and tilt the ROI to fit the area of interest of the skin if necessary.

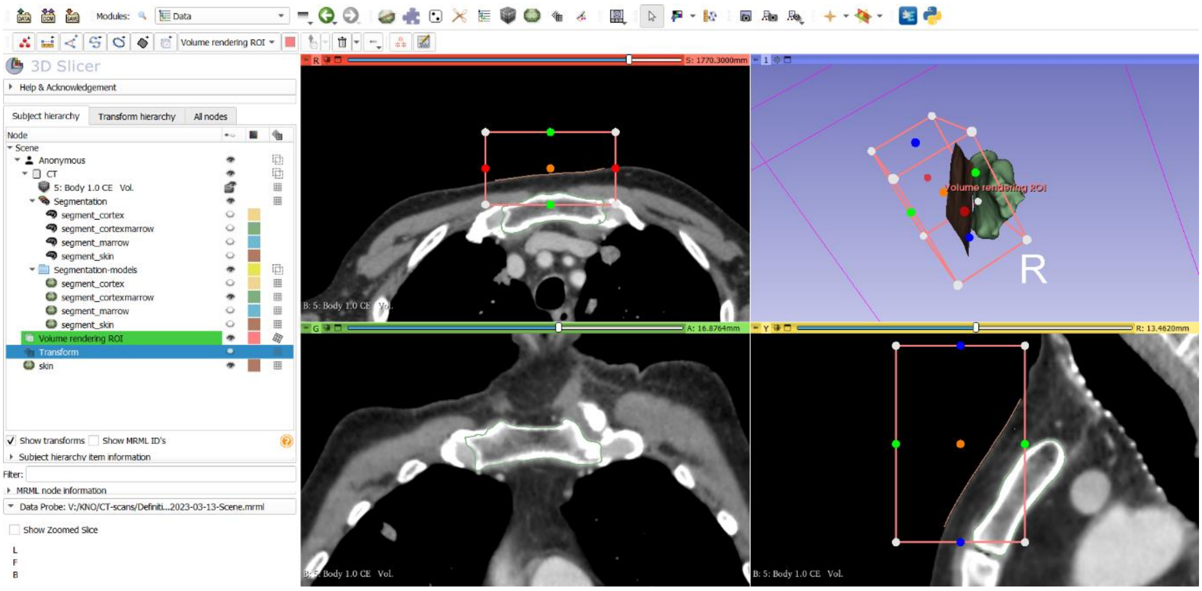

4. **Extra measurements**
   - Bone density was measured using the Segment Statistics module.
   - The observer reliability scores were obtained using the Segment Comparison module.

Rotation code

```
clc, clear all, close all

%% Find location data
CM_data = dir('directory name');
M_data = dir('directory name');
S_data = dir('directory name');

for patient = 1:length(CM_data)
disp(['*Patient Nr:' num2str(patient)])

% Load data patient
CM = stlread([CM_data(patient).folder '\' CM_data(patient).name]);
M = stlread([M_data(patient).folder '\' M_data(patient).name]);
Sc = stlread([S_data(patient).folder '\' S_data(patient).name]);

% Set Points to origin
Transform(1,:,patient) = [mean(CM.Points(:,1)), mean(CM.Points(:,2)), mean(CM.Points(:,3))];
CM_origin = CM.Points - Transform(:,:,patient);
M_origin = M.Points - Transform(:,:,patient);
S_origin = Sc.Points - Transform(:,:,patient);

%% Rotation around X-axis
% Create center (diagonal) line
[~,S,V] = svd(CM_origin(:,2:3),'econ');
varS = diag(S);
disp(['Variance PC1 (for rotation in the YZ-plane) is: ' num2str(varS(1)/sum(diag(S)))]);
dir_vec_preX = [0 V(:,1)'];

% Compute angle to y-axis before rotation
X_axis = [1 0 0];
Y_axis = [0 1 0];
CosTheta_preX = max(min(dot(dir_vec_preX,Y_axis)/(norm(dir_vec_preX)*norm(Y_axis)),1),-1);
theta_preX = real(acosd(CosTheta_preX));

% Display the value of the angle before rotation between the diagonal line and the Y-axis
disp(['Before rotation, the angle between the diagonal line and the Y-axis is ', num2str(theta_preX), '
degrees.']);

% Define rotation matrix X
rotationX(:,:,patient) = [1 0 0;...
            0 cosd(theta_preX), -sind(theta_preX);...
            0, sind(theta_preX) cosd(theta_preX)];

% Apply rotation matrix X
CM_rotX = CM_origin*rotationX(:,:,patient);
dir_vec_postX = dir_vec_preX*rotationX(:,:,patient);
M_rotX = M_origin*rotationX(:,:,patient);
S_rotX = S_origin*rotationX(:,:,patient);

% Plot mesh graph w/ vectors after rotation
% Plot original data
hold on
fig1=figure(1),
view (90,0),
plot3(CM_origin(:,1), CM_origin(:,2), CM_origin(:,3),'g.','MarkerSize',0.2);
```

```
green1 = plot3([0 dir_vec_preX(1)*30], [0 dir_vec_preX(2)*30],[0
dir_vec_preX(3)*30],'g','LineWidth',2);
alpha(0.1);
plot3(CM_rotX(:,1),CM_rotX(:,2),CM_rotX(:,3),'r.','MarkerSize',4)
red1 = plot3([0 dir_vec_postX(1)*30] ,[0 dir_vec_postX(2)*30] ,[0
dir_vec_postX(3)*30],'r','LineWidth',2);
axis equal
xlabel('X'), ylabel('Y'), zlabel('Z')
title('A'),fontsize(22,"points")
axis([-40 40 -40 40 -40 40]); % Set ranges for x, y, and z axes
legend ([green1, red1], {'Before rotation', 'After rotation'},'Location','Best')

% Display the value of the angle after rotation between the diagonal line and the Y-axis
CosTheta_postX = max(min(dot(dir_vec_postX,Y_axis)/(norm(dir_vec_postX)*norm(Y_axis)),1),-1);
theta_postX = real(acosd(CosTheta_postX));
disp(['After rotation, the angle between the diagonal line and the Y-axis is ', num2str(theta_postX), '
degrees.']);

%% Rotation around Y-axis
% Create center (horizontal) line
[~,S,V] = svd(CM_rotX(:,1:2:3),'econ');
varS = diag(S);
disp(['Variance PC2 (for rotation in the XZ plane) is: ' num2str(varS(1)/sum(diag(S)))]);
if V(2,1)<0 && V(1,1)>0
  dir_vec_preY = [abs(V(1,1)), 0, V(2,1)];
elseif V(2,1)<0 && V(1,1)<0
  dir_vec_preY = abs([V(1,1), 0, V(2,1)]);
elseif V(2,1)>0 && V(1,1)<0
  dir_vec_preY = [abs(V(1,1)), 0, -V(2,1)];
else
   dir_vec_preY=[V(1,1), 0, V(2,1)];
end

% Compute angle to x-axis before rotation
CosTheta_preY = max(min(dot(dir_vec_preY,X_axis)/(norm(dir_vec_preY)*norm(X_axis)),1),-1);
theta_preY = real(acosd(CosTheta_preY));

% Display the value of the angle before rotation between the horizontal line and the X-axis
disp(['Before rotation, the angle between the horizontal line and the X-axis is ', num2str(theta_preY), '
degrees.']);

% Define rotation matrix Y
if V(2,1) <0 && V(1,1)>0
rotationY(:,:,patient) = [cosd(theta_preY), 0, sind(theta_preY);...
            0, 1, 0;...
            -sind(theta_preY), 0, cosd(theta_preY)];
elseif V(2,1)>0 && V(1,1)<0
    rotationY(:,:,patient) = [cosd(theta_preY), 0, sind(theta_preY);...
            0, 1, 0;...
            -sind(theta_preY), 0, cosd(theta_preY)];
else
    rotationY(:,:,patient) = [cosd(-theta_preY), 0, sind(-theta_preY);...
            0, 1, 0;...
            sind(-theta_preY), 0, cosd(-theta_preY)];
end

% Apply rotation matrix Y
CM_rotXY = CM_rotX*rotationY(:,:,patient);
```

```
dir_vec_postY = dir_vec_preY*rotationY(:,:,patient);
M_rotXY = M_rotX*rotationY(:,:,patient);
S_rotXY = S_rotX*rotationY(:,:,patient);

% Plot mesh graph w/ vectors after rotation around X and Y axes
fig2=figure(2),
hold on
view(0,0),
plot3(CM_rotX(:,1),CM_rotX(:,2),CM_rotX(:,3),'g.','MarkerSize',0.2);
green2 = plot3([0 dir_vec_preY(1)*30] ,[0 dir_vec_preY(2)*30] ,[0
dir_vec_preY(3)*30],'g','LineWidth',2);
plot3(CM_rotXY(:,1),CM_rotXY(:,2),CM_rotXY(:,3),'r.','MarkerSize',4)
red2 = plot3([0 dir_vec_postY(1)*30], [0 dir_vec_postY(2)*30],[0
dir_vec_postY(3)*30],'r','LineWidth',2);
xlabel('X'), ylabel('Y'), zlabel('Z')
axis equal
title('B'),fontsize(22,"points")
axis([-40 40 -40 40 -40 40]); % Set ranges for x, y, and z axes

% Display the value of the angle after rotation between the horizontal line and the X-axis
CosTheta_postY = max(min(dot(dir_vec_postY,X_axis)/(norm(dir_vec_postY)*norm(X_axis)),1),-1);
theta_postY = real(acosd(CosTheta_postY));
disp(['After rotation, the angle between the horizontal line and the X-axis is ', num2str(theta_postY), '
degrees.']);

%% Rotation around Z-axis
% Create line along width from Xmin to Xmax ('latitude line')
[sortedX, sortedIndices] = sort(CM_rotXY(:,1));
percentile = 5; range = 1;
perc = [percentile*0.01, (percentile+range)*0.01]; %select all values between 5th and 6th percentiles
lowestXIndices = sortedIndices([floor(length(sortedX)*perc(1)) : ceil(length(sortedX)*perc(2))] );
highestXIndices = sortedIndices([floor(length(sortedX)*(1-perc(2))) : ceil(length(sortedX)*(1-
perc(1)))]);
minXCoord = CM_rotXY(lowestXIndices,:);
minXCoord_mean = mean(minXCoord);
maxXCoord = CM_rotXY(highestXIndices,:);
maxXCoord_mean = mean(maxXCoord);

if maxXCoord_mean(:,2)>minXCoord_mean(:,2)
dir_vec_preZ = maxXCoord_mean-minXCoord_mean; dir_vec_preZ(3) = 0; dir_vec_preZ =
(dir_vec_preZ/norm(dir_vec_preZ));
else
dir_vec_preZ = minXCoord_mean-maxXCoord_mean; dir_vec_preZ(3) = 0; dir_vec_preZ = -
(dir_vec_preZ/norm(dir_vec_preZ));
end

% Compute angle before rotation
if minXCoord_mean(:,2)<maxXCoord_mean(:,2)
CosTheta_preZ = max(min(dot(dir_vec_preZ,X_axis)/(norm(dir_vec_preZ)*norm(X_axis)),1),-1);
theta_preZ = real(acosd(CosTheta_preZ));
rotationZ(:,:,patient) = [cosd(theta_preZ), -sind(theta_preZ), 0 ;...
            sind(theta_preZ), cosd(theta_preZ) 0;...
            0, 0, 1];
else
CosTheta_preZ = max(min(dot(dir_vec_preZ,X_axis)/(norm(dir_vec_preZ)*norm(X_axis)),1),-1);
theta_preZ = real(acosd(CosTheta_preZ));
rotationZ(:,:,patient) = [cosd(-theta_preZ), -sind(-theta_preZ), 0 ;...
            sind(-theta_preZ), cosd(-theta_preZ) 0;...
```

```
            0, 0, 1];
end

% Display the value of the angle before rotation between the latitude line and the X-axis
disp(['Before rotation, the angle between the latitude line and the X-axis is ', num2str(theta_preZ), '
degrees.']);

% Apply rotation matrix Z
CM_rotXYZ = CM_rotXY*rotationZ(:,:,patient);
dir_vec_postZ = dir_vec_preZ*rotationZ(:,:,patient);
M_rotXYZ = M_rotXY*rotationZ(:,:,patient);
S_rotXYZ = S_rotXY*rotationZ(:,:,patient);

% Plot mesh graph w/ vectors after rotation around XYZ axes
fig3=figure(3);
hold on
view(0,90),
plot3(CM_rotXY(:,1),CM_rotXY(:,2),CM_rotXY(:,3),'g.','MarkerSize',0.2)
green3 = plot3([0 dir_vec_preZ(1)*30],[0 dir_vec_preZ(2)*30], [0
dir_vec_preZ(3)*30],'g','LineWidth',2);
plot3(minXCoord(:,1),minXCoord(:,2),minXCoord(:,3),'b.','MarkerSize',5)
plot3(minXCoord_mean(1),minXCoord_mean(2),minXCoord_mean(3),'c*','MarkerSize',5)
plot3(maxXCoord(:,1),maxXCoord(:,2),maxXCoord(:,3),'b.','MarkerSize',5)
plot3(maxXCoord_mean(1),maxXCoord_mean(2),maxXCoord_mean(3),'c*','MarkerSize',5)
plot3(CM_rotXYZ(:,1),CM_rotXYZ(:,2),CM_rotXYZ(:,3),'r.','MarkerSize',4)
red3 = plot3([0 dir_vec_postZ(1)*30] ,[0 dir_vec_postZ(2)*30] ,[0
dir_vec_postZ(3)*30],'r','LineWidth',2);
xlabel('X'), ylabel('Y'), zlabel('Z')
axis equal
title('C'),fontsize(22,"points")
axis([-40 40 -40 40 -40 40]); % Set ranges for x, y, and z axes

% Display the value of the angle after rotation between the latitude line and the X-axis
CosTheta_postZ = max(min(dot(dir_vec_postZ,X_axis)/(norm(dir_vec_postZ)*norm(X_axis)),1),-1);
theta_postZ = real(acosd(CosTheta_postZ));
disp(['After rotation, the angle between the latitude line and the X-axis is ', num2str(theta_postZ), '
degrees.']);

list_ptRot = [1 4 11 21 22 26 27 30 32 34 35 39 46 49];
list_ptAngles = [5 -5 5 -5 -5 5 8 -8 5 -5 -5 10 -5 -10];
rotationZ_2(:,:,patient) = eye(3);
if any(patient == list_ptRot)
   ind = find(patient==list_ptRot);
   angle = list_ptAngles(ind);
   alpha = deg2rad(angle);
   rotationZ_2(:,:,patient) = [cos(alpha) -sin(alpha) 0; sin(alpha) cos(alpha) 0; 0 0 1];
   CM_rotXYZ=CM_rotXYZ*rotationZ_2(:,:,patient);
   M_rotXYZ = M_rotXYZ*rotationZ_2(:,:,patient);
   S_rotXYZ = S_rotXYZ*rotationZ_2(:,:,patient);
end
%% Automatic jugular notch selection based on highest Z-coordinates with neg/pos X-coordinates
CM_rotXYZ_x = CM_rotXYZ(:,1);
CM_rotXYZ_y = CM_rotXYZ(:,2);
CM_rotXYZ_boundary = boundary(CM_rotXYZ_x,CM_rotXYZ_y); %find edge
boundary_points = [CM_rotXYZ_x(CM_rotXYZ_boundary) CM_rotXYZ_y(CM_rotXYZ_boundary)];
boundary_points_x = boundary_points(:,2);
A = find(boundary_points_x>0);
boundary_points_sup = boundary_points(A,:);
```

```
B = find(CM_rotXYZ(:,1)>0);
CM_rotXYZ_pos = CM_rotXYZ(B,:);
C = find (CM_rotXYZ(:,1)<0);
CM_rotXYZ_neg = CM_rotXYZ(C,:);
[a pos_max_x]=max(CM_rotXYZ_pos(:,2));
[b neg_max_x]=max(CM_rotXYZ_neg(:,2));
CM_rotXYZ_pos_max=CM_rotXYZ_pos(pos_max_x,:);
CM_rotXYZ_neg_max=CM_rotXYZ_neg(neg_max_x,:);
CM_rotXYZ_mean=mean([CM_rotXYZ_pos_max(:,1) CM_rotXYZ_neg_max(:,1)]);

distances_to_mid=abs(boundary_points_sup(:,1) - CM_rotXYZ_mean);
closest_to_zero=find(distances_to_mid == min(distances_to_mid));

figure('units','normalized','outerposition',[0 0 1 1])
hold on
scatter(CM_rotXYZ_x,CM_rotXYZ_y,1,'g','filled')
scatter(boundary_points(:,1),boundary_points(:,2),1,'r')
scatter(boundary_points_sup(closest_to_zero,1),boundary_points_sup(closest_to_zero,2),15,'k','filled
')
scatter(CM_rotXYZ_pos_max(:,1),CM_rotXYZ_pos_max(:,2),15,'b','filled')
scatter(CM_rotXYZ_neg_max(:,1),CM_rotXYZ_neg_max(:,2),15,'b','filled')
title('D'),fontsize(22,"points")
xlabel('X'), ylabel('Y'), zlabel('Z')
axis equal
axis([-40 40 -40 40 -40 40]); % Set ranges for x, y, and z axes

%% Manual jugular notch selection
list_ptNotch = [2 5 11 12 15 18 21 27 33 36 38 48];
if any(patient == list_ptNotch)

x_line = 0;
y_line = linspace(-30, 30, 100);  % Adjust the number of points as needed
z_line = linspace(-30, 30, 100);  % Adjust the number of points as needed

figure
hold on
view(0,90),
plot3(CM_rotXYZ(:,1),CM_rotXYZ(:,2),CM_rotXYZ(:,3),'g.','MarkerSize',4)
plot3(x_line * ones(size(y_line)), y_line, z_line, 'b', 'LineWidth', 1);
xlabel('X'), ylabel('Y'), zlabel('Z')
axis equal
axis([-40 40 -40 40 -40 40]); % Set ranges for x, y, and z axes
title('E'),fontsize(22,"points")
[x y] = ginput(1);
distances = sqrt((CM_rotXYZ(:, 1) - x).^2 + (CM_rotXYZ(:, 2) - y).^2);
[~, index] = min(distances);

else
   x = boundary_points_sup(closest_to_zero,1);
   y = boundary_points_sup(closest_to_zero,2);
   distances = sqrt((CM_rotXYZ(:, 1) - x).^2 + (CM_rotXYZ(:, 2) - y).^2);
   [~, index] = min(distances);
end

%% Visual check
figure
hold on
```

```
view(0,90)
plot3(CM_rotXYZ(:,1),CM_rotXYZ(:,2),CM_rotXYZ(:,3),'g.','MarkerSize',4)
selectedPoint = CM_rotXYZ(index, :);
plot3(CM_rotXYZ(index,1),CM_rotXYZ(index,2),CM_rotXYZ(index,3),'b.','MarkerSize',20);
xlabel('X'), ylabel('Y'), zlabel('Z')
axis equal
axis([-40 40 -40 40 -40 40]); % Set ranges for x, y, and z axes
title('F'),fontsize(22,"points")
pause

close all
neworigin(:,:,patient) = [CM_rotXYZ(index,1),CM_rotXYZ(index,2),CM_rotXYZ(index,3)];

%% STL file generation
CM_transformed = CM;
M_transformed = M;
S_transformed = Sc;

CM_transformed = CM_transformed.Points - Transform(:,:,patient);
CM_transformed = CM_transformed * rotationX(:,:,patient) * rotationY(:,:,patient) *
rotationZ(:,:,patient) * rotationZ_2(:,:,patient);
CM_transformed = CM_transformed - neworigin(:,:,patient);
CM_new = triangulation(CM.ConnectivityList,CM_transformed);

M_transformed = M_transformed.Points - Transform(:,:,patient);
M_transformed = M_transformed * rotationX(:,:,patient) * rotationY(:,:,patient) * rotationZ(:,:,patient) *
rotationZ_2(:,:,patient);
M_transformed = M_transformed - neworigin(:,:,patient);
M_new = triangulation(M.ConnectivityList,M_transformed);

S_transformed = S_transformed.Points - Transform(:,:,patient);
S_transformed = S_transformed* rotationX(:,:,patient) * rotationY(:,:,patient) * rotationZ(:,:,patient) *
rotationZ_2(:,:,patient);
S_transformed = S_transformed- neworigin(:,:,patient);
S_new = triangulation(Sc.ConnectivityList,S_transformed);

fn = ['patient' num2str(patient)];
stlwrite(CM_new, ['Cortex_marrow (output)' fn '.stl']);
stlwrite(M_new, ['Marrow (output)' fn '.stl']);
stlwrite(S_new, ['Subcutis (output)' fn '.stl']);
end
```

Soft tissue thickness heat map code

```
clc, clear all, close all
figure_on = false;

reference = dir('directory name');
target = dir('directory name');

num_patients=49;

%Heat map size, resolution and angle
minX = -50; maxX = 50; minY = -80; maxYtemp = 20;
res_given = 1;
hoek_toegestaan = 15;
numberRes = ceil((abs(minX)+maxX)/res_given); res = (abs(minX)+maxX)/numberRes;
number_Y = ceil((abs(minY)+maxYtemp)/res); maxY = minY + (number_Y*res);
raster = zeros((abs(minX)+maxX)/res,(abs(minY)+maxY)/res);
raster_manubrium = raster;
raster_subcutis = raster;

tic
for patient = 1:num_patients
clearvars -except raster_averages_angles_weighted raster raster_imp12 raster_imp8 raster_imp6
raster_theta6 raster_manubrium num_patients patient res minX minY reference target figure_on
hoek_toegestaan raster_subcutis
disp(['*Patient Nr:' num2str(patient)])

% Load data patient
CM = stlread([reference(patient).folder '\' reference(patient).name]);
M = stlread([target(patient).folder '\' target(patient).name]);

%% Identifying the margins
% Posterior margin of the anterior cortex & the anterior margin of the...
% posterior cortex (marrow)
indexPMV = find(M.Points(:,3)>0);

checkVoorMerg = sum(ismember(M.ConnectivityList,indexPMV),2) ;
indMergFacesVoor = find(checkVoorMerg==3);
MVoor = triangulation(M.ConnectivityList(indMergFacesVoor,:),M.Points);

if figure_on
hold on,
trisurf(MVoor,'Facecolor','g','FaceAlpha',0.5,'EdgeAlpha',0.1), axis equal
end

% Anterior margin of the anterior cortex & the posterior margin of the...
% posterior cortex (cortex_marrow)
indexPCV = find(CM.Points(:,3)>0);

checkVoorCortex = sum(ismember(CM.ConnectivityList,indexPCV),2) ;
indCortexFacesVoor = find(checkVoorCortex==3);
indCortexFacesAchter = find(checkVoorCortex<3);
CVoor = triangulation(CM.ConnectivityList(indCortexFacesVoor,:),CM.Points);
CAchter = triangulation(CM.ConnectivityList(indCortexFacesAchter,:),CM.Points);

if figure_on
hold on,
trisurf(CVoor,'Facecolor','y','FaceAlpha',0.5,'EdgeAlpha',0.1), axis equal
```

```
trisurf(CAchter,'Facecolor','r','FaceAlpha',0.5,'EdgeAlpha',0.1), axis equal
end

Vertex1 = MVoor.Points(MVoor.ConnectivityList(:,1),:);
Vertex2 = MVoor.Points(MVoor.ConnectivityList(:,2),:);
Vertex3 = MVoor.Points(MVoor.ConnectivityList(:,3),:);

%Distances for faces
Centers= incenter(CVoor); Normals = -faceNormal(CVoor);
thetas = abs(acosd(abs(Normals(:,3))));
for i = 1:length(CVoor.ConnectivityList)
    clear t intersect
    [intersect, t] = TriangleRayIntersection (...
        Centers(i,:), -Normals(i,:), Vertex1, Vertex2, Vertex3);
    if find(intersect)
        distances = t(intersect); non_zeros = t(intersect) ~= 0;
        [~,ii] = min(distances(non_zeros));
         distance_faces(i) = distances(non_zeros(ii));
    else
        distance_faces(i) = 0;
    end
end

%% Heat map
for xi = 1:size(raster,1)
    for yi = 1:size(raster,2)
        ind = find(Centers(:,1) >= minX+(res*(xi-1)) & Centers(:,1) < minX+res+(res*(xi-1)) ...
        &  Centers(:,2) >= minY+(res*(yi-1)) & Centers(:,2) < minY+res +(res*(yi-1)));

        if any(ind)
            raster_manubrium(xi,yi) =  raster_manubrium(xi,yi)+1;
            if any(distance_faces(ind))
                raster_subcutis(xi,yi,patient) = mean(nonzeros(distance_faces(ind)));
            end
        end
    end

end
end
for i = 1:num_patients
    average_ST(i) = mean(nonzeros(raster_subcutis(:,:,i)));

end
average_ST
minimal_ST = min(average_ST), maximal_ST = max(average_ST)
hist(average_ST)

for xi = 1:size(raster,1)
    for yi = 1:size(raster,2)
        rasterSubcutis_average(xi,yi) = mean(nonzeros(raster_subcutis(xi,yi,:)));
        %cap at x
        cap = 30;
        rasterSubcutis_capped = rasterSubcutis_average;
        if any(rasterSubcutis_capped(:)>cap)
            ind = find(rasterSubcutis_capped>cap);
            rasterSubcutis_capped(ind) = cap;
        end
    end
```

```
end
%% Visualization
figure
imshow(imrotate(rasterSubcutis_capped,90),[]), colormap('jet'), colorbar('XTick',0:5:30),
fontsize(20,"points"), title('D');

%% Mark thickest points
% find(max(perc_imp6))


function [Nx,Ny,Nz,Normal]=patchnormals_double(Fa,Fb,Fc,Vx,Vy,Vz)
%
%  [Nx,Ny,Nz]=patchnormals_double(Fa,Fb,Fc,Vx,Vy,Vz)
%
FV.vertices=zeros(length(Vx),3);
FV.vertices(:,1)=Vx;
FV.vertices(:,2)=Vy;
FV.vertices(:,3)=Vz;
% Get all edge vectors
e1=FV.vertices(Fa,:)-FV.vertices(Fb,:);
e2=FV.vertices(Fb,:)-FV.vertices(Fc,:);
e3=FV.vertices(Fc,:)-FV.vertices(Fa,:);
% Normalize edge vectors
e1_norm=e1./repmat(sqrt(e1(:,1).^2+e1(:,2).^2+e1(:,3).^2),1,3);
e2_norm=e2./repmat(sqrt(e2(:,1).^2+e2(:,2).^2+e2(:,3).^2),1,3);
e3_norm=e3./repmat(sqrt(e3(:,1).^2+e3(:,2).^2+e3(:,3).^2),1,3);
% Calculate Angle of face seen from vertices
Angle =  [acos(dot(e1_norm',-e3_norm'));acos(dot(e2_norm',-e1_norm'));acos(dot(e3_norm',-
e2_norm'))]';
% Calculate normal of face
Normal=cross(e1,e3);

% Calculate Vertice Normals
VerticeNormals=zeros([size(FV.vertices,1) 3]);
for i=1:size(Fa,1)
    VerticeNormals(Fa(i),:)=VerticeNormals(Fa(i),:)+Normal(i,:)*Angle(i,1);
    VerticeNormals(Fb(i),:)=VerticeNormals(Fb(i),:)+Normal(i,:)*Angle(i,2);
    VerticeNormals(Fc(i),:)=VerticeNormals(Fc(i),:)+Normal(i,:)*Angle(i,3);
end
V_norm=sqrt(VerticeNormals(:,1).^2+VerticeNormals(:,2).^2+VerticeNormals(:,3).^2)+eps;
VerticeNormals=VerticeNormals./repmat(V_norm,1,3);
Nx=VerticeNormals(:,1);
Ny=VerticeNormals(:,2);
Nz=VerticeNormals(:,3);
end


function [intersect, t, u, v, xcoor] = TriangleRayIntersection (...
  orig, dir, vert0, vert1, vert2, varargin)

% Transpose inputs if needed
if (size(orig ,1)==3 && size(orig ,2)~=3), orig =orig' ; end
if (size(dir  ,1)==3 && size(dir  ,2)~=3), dir  =dir'  ; end
if (size(vert0,1)==3 && size(vert0,2)~=3), vert0=vert0'; end
if (size(vert1,1)==3 && size(vert1,2)~=3), vert1=vert1'; end
if (size(vert2,1)==3 && size(vert2,2)~=3), vert2=vert2'; end
% In case of single points clone them to the same size as the rest
N = max([size(orig,1), size(dir,1), size(vert0,1), size(vert1,1), size(vert2,1)]);
```

```
if (size(orig ,1)==1 && N>1 && size(orig ,2)==3), orig  = repmat(orig , N, 1); end
if (size(dir  ,1)==1 && N>1 && size(dir  ,2)==3), dir   = repmat(dir  , N, 1); end
if (size(vert0,1)==1 && N>1 && size(vert0,2)==3), vert0 = repmat(vert0, N, 1); end
if (size(vert1,1)==1 && N>1 && size(vert1,2)==3), vert1 = repmat(vert1, N, 1); end
if (size(vert2,1)==1 && N>1 && size(vert2,2)==3), vert2 = repmat(vert2, N, 1); end
% Check if all the sizes match
SameSize = (any(size(orig)==size(vert0)) && ...
  any(size(orig)==size(vert1)) && ...
  any(size(orig)==size(vert2)) && ...
  any(size(orig)==size(dir  )) );
assert(SameSize && size(orig,2)==3, ...
  'All input vectors have to be in Nx3 format.');
% Read user preferences
eps        = 1e-5;
planeType  = 'two sided';
lineType   = 'ray';
border     = 'normal';
fullReturn = false;
nVarargs   = length(varargin);
k = 1;
if nVarargs>0 && isstruct(varargin{1})
  % This section is provided for backward compability only
  options = varargin{1};
  if (isfield(options, 'eps'     )), eps       = options.eps;      end
  if (isfield(options, 'triangle')), planeType= options.triangle; end
  if (isfield(options, 'ray'     )), lineType = options.ray;      end
  if (isfield(options, 'border'  )), border   = options.border;   end
else
  while (k<=nVarargs)
    assert(ischar(varargin{k}), 'Incorrect input parameters')
    switch lower(varargin{k})
      case 'eps'
        eps = abs(varargin{k+1});
        k = k+1;
      case 'planetype'
        planeType = lower(strtrim(varargin{k+1}));
        k = k+1;
      case 'border'
        border = lower(strtrim(varargin{k+1}));
        k = k+1;
      case 'linetype'
        lineType = lower(strtrim(varargin{k+1}));
        k = k+1;
      case 'fullreturn'
        fullReturn = (double(varargin{k+1})~=0);
        k = k+1;
    end
    k = k+1;
  end
end
% Set up border parameter
switch border
  case 'normal'
    zero=0.0;
  case 'inclusive'
    zero=eps;
  case 'exclusive'
    zero=-eps;
```

```
  otherwise
    error('Border parameter must be either "normal", "inclusive" or "exclusive"')
end
%% initialize default output
intersect = false(size(orig,1),1); % by default there are no intersections
t = inf+zeros(size(orig,1),1); u=t; v=t;
xcoor = nan+zeros(size(orig));
%% Find faces parallel to the ray
edge1 = vert1-vert0;          % find vectors for two edges sharing vert0
edge2 = vert2-vert0;
tvec  = orig -vert0;          % vector from vert0 to ray origin
pvec  = cross(dir, edge2,2);  % begin calculating determinant - also used to calculate U parameter
det   = sum(edge1.*pvec,2);   % determinant of the matrix M = dot(edge1,pvec)
switch planeType
  case 'two sided'            % treats triangles as two sided
    angleOK = (abs(det)>eps); % if determinant is near zero then ray lies in the plane of the triangle
  case 'one sided'            % treats triangles as one sided
    angleOK = (det>eps);
  otherwise
    error('Triangle parameter must be either "one sided" or "two sided"');
end
if all(~angleOK), return; end % if all parallel than no intersections
%% Different behavior depending on one or two sided triangles
det(~angleOK) = nan;              % change to avoid division by zero
u    = sum(tvec.*pvec,2)./det;    % 1st barycentric coordinate
if fullReturn
  % calculate all variables for all line/triangle pairs
  qvec = cross(tvec, edge1,2);    % prepare to test V parameter
  v    = sum(dir  .*qvec,2)./det; % 2nd barycentric coordinate
  t    = sum(edge2.*qvec,2)./det; % 'position on the line' coordinate
  % test if line/plane intersection is within the triangle
  ok   = (angleOK & u>=-zero & v>=-zero & u+v<=1.0+zero);
else
  % limit some calculations only to line/triangle pairs where it makes
  % a difference. It is tempting to try to push this concept of
  % limiting the number of calculations to only the necessary to "u"
  % and "t" but that produces slower code
  v = nan+zeros(size(u)); t=v;
  ok = (angleOK & u>=-zero & u<=1.0+zero); % mask
  % if all line/plane intersections are outside the triangle than no intersections
  if ~any(ok), intersect = ok; return; end
  qvec = cross(tvec(ok,:), edge1(ok,:),2); % prepare to test V parameter
  v(ok,:) = sum(dir(ok,:).*qvec,2) ./ det(ok,:); % 2nd barycentric coordinate
  if (~strcmpi(lineType,'line')) % 'position on the line' coordinate
    t(ok,:) = sum(edge2(ok,:).*qvec,2)./det(ok,:);
  end
  % test if line/plane intersection is within the triangle
  ok = (ok & v>=-zero & u+v<=1.0+zero);
end
%% Test where along the line the line/plane intersection occurs
switch lineType
  case 'line'      % infinite line
    intersect = ok;
  case 'ray'       % ray is bound on one side
    intersect = (ok & t>=-zero); % intersection on the correct side of the origin
  case 'segment'   % segment is bound on two sides
    intersect = (ok & t>=-zero & t<=1.0+zero); % intersection between origin and destination
  otherwise
```

```
    error('lineType parameter must be either "line", "ray" or "segment"');
end
%% calculate intersection coordinates if requested
if (nargout>4)
      ok = intersect | fullReturn;
      xcoor(ok,:) = vert0(ok,:) ...
        + edge1(ok,:).*repmat(u(ok,1),1,3) ...
        + edge2(ok,:).*repmat(v(ok,1),1,3);
end
end
```

Heat maps for presence of bone tissue, slope and unicortical thickness

```
clc, clear all, close all
figure_on = false;

reference = dir('directory name');
target = dir('directory name');

num_patients=49;

%Heat map size, resolution and angle
minX = -50; maxX = 50; minY = -80; maxYtemp = 20;
res_given = 1;
hoek_toegestaan = 15;
numberRes = ceil((abs(minX)+maxX)/res_given); res = (abs(minX)+maxX)/numberRes;
number_Y = ceil((abs(minY)+maxYtemp)/res); maxY = minY + (number_Y*res);
raster = zeros((abs(minX)+maxX)/res,(abs(minY)+maxY)/res); raster_pat =
[zeros((abs(minX)+maxX)/res,(abs(minY)+maxY)/res,num_patients)];
raster_manubrium = raster;
raster_subcutis = raster;
raster_imp6 = raster;
raster_imp8 = raster;
raster_imp10 = raster;
raster_imp12 = raster;
raster_theta6 = raster;
raster_averages_angles_weighted = raster_pat;
raster_imp6_pat = raster_pat;
raster_imp6_angle = raster_pat;
raster_imp8_pat = raster_pat;
raster_imp8_angle = raster_pat;
raster_imp10_pat = raster_pat;
raster_imp10_angle = raster_pat;
raster_imp12_pat = raster_pat;
raster_imp12_angle = raster_pat;
tic
for patient = 1:num_patients
    clearvars -except raster_averages_angles_weighted raster_pat raster raster_imp6_angle
raster_imp8_angle raster_imp10_angle raster_imp12_angle raster_imp12 raster_imp10 raster_imp8
raster_imp6 raster_imp6_pat raster_imp8_pat raster_imp10_pat raster_imp12_pat raster_theta6
raster_manubrium num_patients patient res minX minY reference target figure_on hoek_toegestaan
raster_subcutis
    disp(['*Patient Nr:' num2str(patient)])

    % Load data patient
    CM = stlread([reference(patient).folder '\' reference(patient).name]);
    M = stlread([target(patient).folder '\' target(patient).name]);

    %% Identifying the margins
    % Posterior margin of the anterior cortex & the anterior margin of the...
    % posterior cortex (marrow)
    indexPMV = find(M.Points(:,3)>0);

    checkVoorMerg = sum(ismember(M.ConnectivityList,indexPMV),2) ;
    indMergFacesVoor = find(checkVoorMerg==3);
    indMergFacesAchter = find(checkVoorMerg<3);
    MVoor = triangulation(M.ConnectivityList(indMergFacesVoor,:),M.Points);
    MAchter = triangulation(M.ConnectivityList(indMergFacesAchter,:),M.Points);
```

```
if figure_on
   hold on,
   trisurf(MVoor,'Facecolor','g','FaceAlpha',0.5,'EdgeAlpha',0.1), axis equal
end

% Anterior margin of the anterior cortex & the posterior margin of the...
% posterior cortex (cortex_marrow)
indexPCV = find(CM.Points(:,3)>0);

checkVoorCortex = sum(ismember(CM.ConnectivityList,indexPCV),2) ;
indCortexFacesVoor = find(checkVoorCortex==3);
indCortexFacesAchter = find(checkVoorCortex<3);
CVoor = triangulation(CM.ConnectivityList(indCortexFacesVoor,:),CM.Points);
CAchter = triangulation(CM.ConnectivityList(indCortexFacesAchter,:),CM.Points);

if figure_on
   hold on,
   trisurf(CVoor,'Facecolor','y','FaceAlpha',0.5,'EdgeAlpha',0.1), axis equal
   trisurf(CAchter,'Facecolor','r','FaceAlpha',0.5,'EdgeAlpha',0.1), axis equal
end

Vertex1 = MAchter.Points(MAchter.ConnectivityList(:,1),:);
Vertex2 = MAchter.Points(MAchter.ConnectivityList(:,2),:);
Vertex3 = MAchter.Points(MAchter.ConnectivityList(:,3),:);

%Distances for faces
Centers= incenter(CVoor); Normals = -faceNormal(CVoor);
thetas = abs(acosd(abs(Normals(:,3))));
for i = 1:length(CVoor.ConnectivityList)
   clear t intersect
   [intersect, t] = TriangleRayIntersection (...
      Centers(i,:), Normals(i,:), Vertex1, Vertex2, Vertex3);

   if find(intersect)
      distances = t(intersect); non_zeros = t(intersect) ~= 0;
      [~,ii] = min(distances(non_zeros));
      distance_faces(i) = distances(non_zeros(ii));
   else
      distance_faces(i) = 0;
   end
end

%% Heat maps
for xi = 1:size(raster,1)
   for yi = 1:size(raster,2)
      ind = find(Centers(:,1) >= minX+(res*(xi-1)) & Centers(:,1) < minX+res+(res*(xi-1)) ...
         &  Centers(:,2) >= minY+(res*(yi-1)) & Centers(:,2) < minY+res +(res*(yi-1)));
      all_data_rasterpoint = thetas(ind)';
      raster_averages_angles_weighted(xi,yi,patient) = mean(all_data_rasterpoint);

      if any(ind)
         raster_manubrium(xi,yi) =  raster_manubrium(xi,yi)+1;
         if any(distance_faces(ind))
            raster_subcutis(xi,yi,patient) = mean(nonzeros(distance_faces(ind)));
         end
      end

      if any(thetas(ind)<hoek_toegestaan)
```

```
                raster_theta6(xi,yi) = raster_theta6(xi,yi)+1;
            end
            if ~any(distance_faces(ind))
                raster_subcutis(xi,yi) = raster_subcutis(xi,yi)+1;
            end
            if any(distance_faces(ind)>12)
                raster_imp12_pat(xi,yi,patient) = 1;
                raster_imp12(xi,yi) = raster_imp12(xi,yi)+1;
            end
            if any(distance_faces(ind)>10)
                raster_imp10_pat(xi,yi,patient) = 1;
                raster_imp10(xi,yi) = raster_imp10(xi,yi)+1;
            end
            if any(distance_faces(ind)>8)
                raster_imp8_pat(xi,yi,patient) = 1;
                raster_imp8(xi,yi) = raster_imp8(xi,yi)+1;
            end
            if any(distance_faces(ind)>6)
                raster_imp6_pat(xi,yi,patient) = 1;
                raster_imp6(xi,yi) = raster_imp6(xi,yi)+1;
            end
        end
    end
end
for i = 1:num_patients
    [r6,c6] = find(raster_imp6_pat(:,:,i) & raster_averages_angles_weighted(:,:,i)<=15);
    [r8,c8] = find(raster_imp8_pat(:,:,i) & raster_averages_angles_weighted(:,:,i)<=15);
    [r10,c10] = find(raster_imp10_pat(:,:,i) & raster_averages_angles_weighted(:,:,i)<=15);
    [r12,c12] = find(raster_imp12_pat(:,:,i) & raster_averages_angles_weighted(:,:,i)<=15);

    for j = 1:length(r6)
        raster_imp6_angle(r6(j),c6(j),i) = 1;
    end
    for j = 1:length(r8)
        raster_imp8_angle(r8(j),c8(j),i) = 1;
    end
    for j = 1:length(r10)
        raster_imp10_angle(r10(j),c10(j),i) = 1;
    end
    for j = 1:length(r12)
        raster_imp12_angle(r12(j),c12(j),i) = 1;
    end
end
%% Visualization

figure
perc_manubrium = raster_manubrium/num_patients;
imshow(imrotate(perc_manubrium,90)), colormap('jet'), title('A'), fontsize(20,"points");

figure
perc_theta6 =  raster_theta6/num_patients;
imshow(imrotate(perc_theta6,90)); colormap('jet'),colorbar('TickLabels', {'0', '20', '40', '60', '80',
'100'}),title('B'), fontsize(20,"points");

figure
imshow(imrotate(mean(raster_averages_angles_weighted,3,"omitnan"),90),[]),colorbar,colormap('jet')
, title('C'), clim([0 45]), fontsize(20,"points");
```

```
figure
perc_imp6 =  raster_imp6/num_patients;
imshow(imrotate(perc_imp6,90)), colormap('jet'), title('E1'), fontsize(20,"points");

figure
perc_imp8 = raster_imp8/num_patients;
imshow(imrotate(perc_imp8,90)), colormap('jet'), title('E2'), fontsize(20,"points");

figure
perc_imp10 = raster_imp10/num_patients;
imshow(imrotate(perc_imp10,90)), colormap('jet'), title('E3'), fontsize(20,"points");

figure
perc_imp12 = raster_imp12/num_patients;
imshow(imrotate(perc_imp12,90)), colormap('jet'), title('E4'), colorbar('TickLabels', {'0', '20', '40', '60',
'80', '100'}), fontsize(20,"points");

figure
showimp6_angle = mean(raster_imp6_angle,3);
imshow(imrotate(showimp6_angle,90)), colormap('jet'), title('F1'),fontsize(20,"points");

figure
showimp8_angle = mean(raster_imp8_angle,3);
imshow(imrotate(showimp8_angle,90)), colormap('jet'), title('F2'),fontsize(20,"points");

figure
showimp10_angle = mean(raster_imp10_angle,3);
imshow(imrotate(showimp10_angle,90)), colormap('jet'), title('F3'), fontsize(20,"points");

figure
showimp12_angle = mean(raster_imp12_angle,3);
imshow(imrotate(showimp12_angle,90)), colormap('jet'), colorbar('TickLabels', {'0', '20', '40', '60', '80',
'100'}), title('F4'), fontsize(20,"points");

%% Mark thickest points
find(max(perc_imp6))

function [Nx,Ny,Nz,Normal]=patchnormals_double(Fa,Fb,Fc,Vx,Vy,Vz)
%
%  [Nx,Ny,Nz]=patchnormals_double(Fa,Fb,Fc,Vx,Vy,Vz)
%
FV.vertices=zeros(length(Vx),3);
FV.vertices(:,1)=Vx;
FV.vertices(:,2)=Vy;
FV.vertices(:,3)=Vz;
% Get all edge vectors
e1=FV.vertices(Fa,:)-FV.vertices(Fb,:);
e2=FV.vertices(Fb,:)-FV.vertices(Fc,:);
e3=FV.vertices(Fc,:)-FV.vertices(Fa,:);
% Normalize edge vectors
e1_norm=e1./repmat(sqrt(e1(:,1).^2+e1(:,2).^2+e1(:,3).^2),1,3);
e2_norm=e2./repmat(sqrt(e2(:,1).^2+e2(:,2).^2+e2(:,3).^2),1,3);
e3_norm=e3./repmat(sqrt(e3(:,1).^2+e3(:,2).^2+e3(:,3).^2),1,3);
% Calculate Angle of face seen from vertices
Angle =  [acos(dot(e1_norm',-e3_norm'));acos(dot(e2_norm',-e1_norm'));acos(dot(e3_norm',-
e2_norm'))]';
% Calculate normal of face
Normal=cross(e1,e3);
```

```
% Calculate Vertice Normals
VerticeNormals=zeros([size(FV.vertices,1) 3]);
for i=1:size(Fa,1)
    VerticeNormals(Fa(i),:)=VerticeNormals(Fa(i),:)+Normal(i,:)*Angle(i,1);
    VerticeNormals(Fb(i),:)=VerticeNormals(Fb(i),:)+Normal(i,:)*Angle(i,2);
    VerticeNormals(Fc(i),:)=VerticeNormals(Fc(i),:)+Normal(i,:)*Angle(i,3);
end
V_norm=sqrt(VerticeNormals(:,1).^2+VerticeNormals(:,2).^2+VerticeNormals(:,3).^2)+eps;
VerticeNormals=VerticeNormals./repmat(V_norm,1,3);
Nx=VerticeNormals(:,1);
Ny=VerticeNormals(:,2);
Nz=VerticeNormals(:,3);
end


function [intersect, t, u, v, xcoor] = TriangleRayIntersection (...
    orig, dir, vert0, vert1, vert2, varargin)

% Transpose inputs if needed
if (size(orig ,1)==3 && size(orig ,2)~=3), orig =orig' ; end
if (size(dir  ,1)==3 && size(dir  ,2)~=3), dir  =dir'  ; end
if (size(vert0,1)==3 && size(vert0,2)~=3), vert0=vert0'; end
if (size(vert1,1)==3 && size(vert1,2)~=3), vert1=vert1'; end
if (size(vert2,1)==3 && size(vert2,2)~=3), vert2=vert2'; end
% In case of single points clone them to the same size as the rest
N = max([size(orig,1), size(dir,1), size(vert0,1), size(vert1,1), size(vert2,1)]);
if (size(orig ,1)==1 && N>1 && size(orig ,2)==3), orig  = repmat(orig , N, 1); end
if (size(dir  ,1)==1 && N>1 && size(dir  ,2)==3), dir   = repmat(dir  , N, 1); end
if (size(vert0,1)==1 && N>1 && size(vert0,2)==3), vert0 = repmat(vert0, N, 1); end
if (size(vert1,1)==1 && N>1 && size(vert1,2)==3), vert1 = repmat(vert1, N, 1); end
if (size(vert2,1)==1 && N>1 && size(vert2,2)==3), vert2 = repmat(vert2, N, 1); end
% Check if all the sizes match
SameSize = (any(size(orig)==size(vert0)) && ...
    any(size(orig)==size(vert1)) && ...
    any(size(orig)==size(vert2)) && ...
    any(size(orig)==size(dir  )) );
assert(SameSize && size(orig,2)==3, ...
    'All input vectors have to be in Nx3 format.');
% Read user preferences
eps        = 1e-5;
planeType  = 'two sided';
lineType   = 'ray';
border     = 'normal';
fullReturn = false;
nVarargs   = length(varargin);
k = 1;
if nVarargs>0 && isstruct(varargin{1})
    % This section is provided for backward compability only
    options = varargin{1};
    if (isfield(options, 'eps'     )), eps      = options.eps;      end
    if (isfield(options, 'triangle')), planeType= options.triangle; end
    if (isfield(options, 'ray'     )), lineType = options.ray;      end
    if (isfield(options, 'border'  )), border   = options.border;   end
else
    while (k<=nVarargs)
        assert(ischar(varargin{k}), 'Incorrect input parameters')
        switch lower(varargin{k})
```

```
            case 'eps'
                eps = abs(varargin{k+1});
                k = k+1;
            case 'planetype'
                planeType = lower(strtrim(varargin{k+1}));
                k = k+1;
            case 'border'
                border = lower(strtrim(varargin{k+1}));
                k = k+1;
            case 'linetype'
                lineType = lower(strtrim(varargin{k+1}));
                k = k+1;
            case 'fullreturn'
                fullReturn = (double(varargin{k+1})~=0);
                k = k+1;
        end
        k = k+1;
    end
end
% Set up border parameter
switch border
    case 'normal'
        zero=0.0;
    case 'inclusive'
        zero=eps;
    case 'exclusive'
        zero=-eps;
    otherwise
        error('Border parameter must be either "normal", "inclusive" or "exclusive"')
end
%% initialize default output
intersect = false(size(orig,1),1); % by default there are no intersections
t = inf+zeros(size(orig,1),1); u=t; v=t;
xcoor = nan+zeros(size(orig));
%% Find faces parallel to the ray
edge1 = vert1-vert0;          % find vectors for two edges sharing vert0
edge2 = vert2-vert0;
tvec  = orig -vert0;          % vector from vert0 to ray origin
pvec  = cross(dir, edge2,2);  % begin calculating determinant - also used to calculate U parameter
det   = sum(edge1.*pvec,2);   % determinant of the matrix M = dot(edge1,pvec)
switch planeType
    case 'two sided'          % treats triangles as two sided
        angleOK = (abs(det)>eps); % if determinant is near zero then ray lies in the plane of the triangle
    case 'one sided'          % treats triangles as one sided
        angleOK = (det>eps);
    otherwise
        error('Triangle parameter must be either "one sided" or "two sided"');
end
if all(~angleOK), return; end % if all parallel than no intersections
%% Different behavior depending on one or two sided triangles
det(~angleOK) = nan;              % change to avoid division by zero
u    = sum(tvec.*pvec,2)./det;    % 1st barycentric coordinate
if fullReturn
    % calculate all variables for all line/triangle pairs
    qvec = cross(tvec, edge1,2);    % prepare to test V parameter
    v    = sum(dir  .*qvec,2)./det; % 2nd barycentric coordinate
    t    = sum(edge2.*qvec,2)./det; % 'position on the line' coordinate
    % test if line/plane intersection is within the triangle
```

```
    ok   = (angleOK & u>=-zero & v>=-zero & u+v<=1.0+zero);
else
    % limit some calculations only to line/triangle pairs where it makes
    % a difference. It is tempting to try to push this concept of
    % limiting the number of calculations to only the necessary to "u"
    % and "t" but that produces slower code
    v = nan+zeros(size(u)); t=v;
    ok = (angleOK & u>=-zero & u<=1.0+zero); % mask
    % if all line/plane intersections are outside the triangle than no intersections
    if ~any(ok), intersect = ok; return; end
    qvec = cross(tvec(ok,:), edge1(ok,:),2); % prepare to test V parameter
    v(ok,:) = sum(dir(ok,:).*qvec,2) ./ det(ok,:); % 2nd barycentric coordinate
    if (~strcmpi(lineType,'line')) % 'position on the line' coordinate
        t(ok,:) = sum(edge2(ok,:).*qvec,2)./det(ok,:);
    end
    % test if line/plane intersection is within the triangle
    ok = (ok & v>=-zero & u+v<=1.0+zero);
end
%% Test where along the line the line/plane intersection occurs
switch lineType
    case 'line'      % infinite line
        intersect = ok;
    case 'ray'       % ray is bound on one side
        intersect = (ok & t>=-zero); % intersection on the correct side of the origin
    case 'segment'   % segment is bound on two sides
        intersect = (ok & t>=-zero & t<=1.0+zero); % intersection between origin and destination
    otherwise
        error('lineType parameter must be either "line", "ray" or "segment"');
end
%% calculate intersection coordinates if requested
if (nargout>4)
    ok = intersect | fullReturn;
    xcoor(ok,:) = vert0(ok,:) ...
        + edge1(ok,:).*repmat(u(ok,1),1,3) ...
        + edge2(ok,:).*repmat(v(ok,1),1,3);
end
end
```

Manual corrections

1. Rotation correction: patients 1 (5°), 4 (-5°), 11 (5°), 21 (-5°), 22 (-5°), 26 (5°), 27 (8°), 30 (-10°), 32 (5°), 34 (-5°), 35 (-5°), 39 (10°), 46 (-5°), 49 (-10°).
2. Jugular notch selection: patients 2, 5, 11, 15, 21, 27, 33, 36 and 38.

Average soft tissue thickness (ST) values

average_ST =

Columns 1 through 18

12.7727 10.7054 20.0023 17.5624 14.4404 17.7460 32.9949 17.3655 13.4953 20.0333 40.7827 11.7455 12.9417 47.2658 22.2723 9.4413 23.1499 8.5575

Columns 19 through 36

7.8067 14.8924 13.8927 38.2117 11.0769 14.9572 14.2578 7.3880 19.4607 28.9652 10.2675 21.9872 27.4390 23.4809 12.7138 22.4146 18.6848 4.4362

Columns 37 through 49

21.0147 7.4050 27.6870 7.9370 15.2833 8.5853 19.1551 10.7392 15.6955 6.7530 16.4137 9.8230 12.4362

minimal_ST =

4.4362

maximal_ST =

47.2658

Histogram showing distribution of average soft tissue thickness values

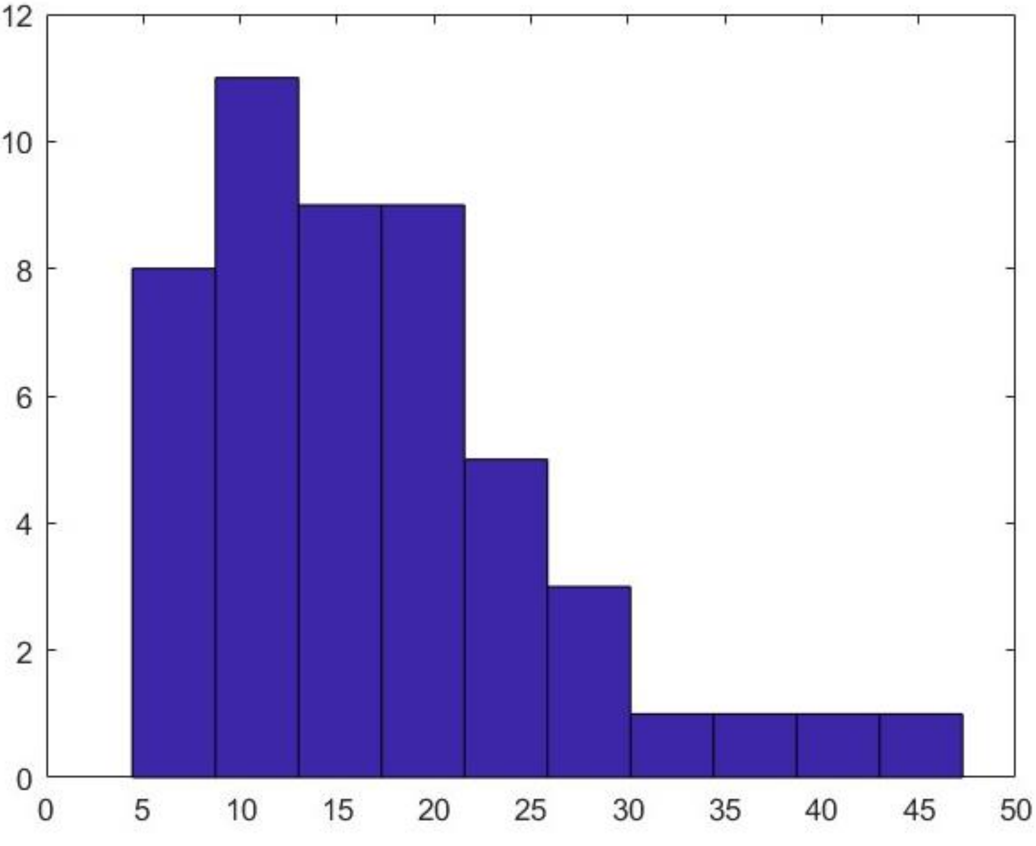

Directed acyclic graph

```
dag {bb="0,0,1,1"
"Body height" [pos="0.048,0.149"]
"Bone classification" [outcome,pos="0.594,0.859"]
"Bone density" [outcome,pos="0.661,0.777"]
"Nutritional status" [latent,pos="0.442,0.053"]
"Oncological treatment" [pos="0.129,0.579"]
"Smoking status" [pos="0.051,0.272"]
"Soft tissue thickness" [outcome,pos="0.727,0.577"]
Age [pos="0.150,0.057"]
BMI [pos="0.213,0.147"]
Diabetes [pos="0.300,0.146"]
Geometry [outcome,pos="0.688,0.687"]
Osteoporosis [pos="0.261,0.767"]
Sex [pos="0.094,0.059"]
Shape [outcome,pos="0.849,0.633"]
Slope [outcome,pos="0.805,0.717"]
"Body height" -> BMI
"Body height" -> Geometry
"Bone density" -> "Bone classification"
"Nutritional status" -> "Bone density"
"Nutritional status" -> "Oncological treatment"
"Nutritional status" -> "Soft tissue thickness"
"Nutritional status" -> BMI [pos="0.323,0.047"]
"Nutritional status" -> Osteoporosis
"Oncological treatment" -> "Bone density"
"Smoking status" -> "Bone density"
Age -> "Oncological treatment"
Age -> "Smoking status"
Age -> BMI
Age -> Diabetes
Age -> Osteoporosis
BMI -> "Bone density"
BMI -> "Oncological treatment"
BMI -> "Soft tissue thickness"
Diabetes -> "Bone density"
Geometry -> Shape
Geometry -> Slope
Osteoporosis -> "Bone density"
Sex -> "Body height"
Sex -> "Smoking status"
Sex -> Geometry [pos="0.384,0.580"]
Sex -> Osteoporosis}
```

Bias & sample size

Selection bias was minimized through random and blinded selection of patients. Measurement bias was minimized by registering scanning parameters, and by having two authors segment the scans. For practical reasons, the measurements were done by one author. As this is a feasibility study, a sample size calculation was not performed, and fifty samples were included to obtain generalizable results.

Possible biasing paths for each exposure of interest

- BMI

None

- Body height

1. Sex

- Sex

None

- Age

None

- Smoking status

1. Age, Sex; 2. BMI, Body height, Diabetes, Nutritional status, Oncological treatment, Osteoporosis, Sex

For each exposure of interest, the most simple biasing paths was chosen to adjust for.

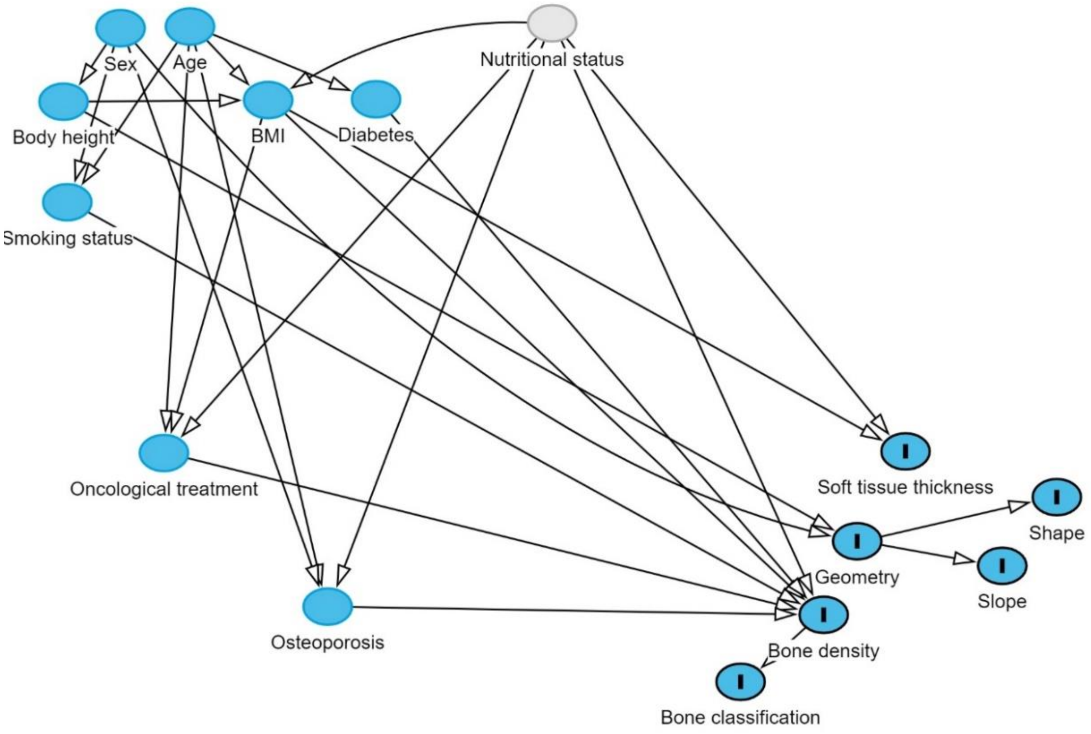
Sex
Age
Nutritional status
Body height
BMI
Diabetes
Smoking status
Oncological treatment
Osteoporosis
Soft tissue thickness
Shape
Geometry
Slope
Bone density
Bone classification

Full statistical syntax

```
* Encoding: UTF-8.

DATASET ACTIVATE DataSet1.

*Creating dummy variables Sex

RECODE Sex ('M'=1) (ELSE=0) INTO Male.

VARIABLE LABELS  Male 'Male'.

EXECUTE.

RECODE Sex ('F'=1) (ELSE=0) INTO Female.

VARIABLE LABELS  Female 'Female'.

EXECUTE.

*Checking mode values for imputation with mode for Smoking status

FREQUENCIES VARIABLES=Smoking_status_string

  /ORDER=ANALYSIS.

*Imputing missing values for Smoking status using the mode

RECODE Smoking_status_string ('Past'=1) ('Current'=2) ('Never'=0) ('Missing'=1) INTO
    Smoking_status_numeric_imputed.

VARIABLE LABELS  Smoking_status_numeric_imputed 'Smoking status numeric'.

EXECUTE.

*Creating dummy variables for Imputed Smoking status

RECODE Smoking_status_numeric_imputed (0=1) (ELSE=0) INTO Smoking_status_never.

VARIABLE LABELS  Smoking_status_never 'Non-smokers'.

EXECUTE.

RECODE Smoking_status_numeric_imputed (1=1) (ELSE=0) INTO Smoking_status_past.

VARIABLE LABELS  Smoking_status_past 'Former smokers'.

EXECUTE.

RECODE Smoking_status_numeric_imputed (2=1) (ELSE=0) INTO Smoking_status_current.

VARIABLE LABELS  Smoking_status_current 'Current smokers'.

EXECUTE.

*Imputing missing values for BMI using the mean

RMV /BMI=SMEAN(BMI).

*Normality check

FREQUENCIES VARIABLES=Age Body_height Soft_tissue_thickness MSB_height
MSB_thickness_thickest

    MSB_thickness_thinnest MSB_width_thickest MSB_width_thinnest Median_density_cortical

    Median_density_cancellous BMI
```

```
  /FORMAT=NOTABLE
  /HISTOGRAM NORMAL
  /ORDER=ANALYSIS.
*Calculating bootstrapped CI intervals
BOOTSTRAP
  /SAMPLING METHOD=SIMPLE
  /VARIABLES TARGET=Age Body_height Soft_tissue_thickness MSB_height
MSB_thickness_thickest
    MSB_thickness_thinnest MSB_width_thickest MSB_width_thinnest Median_density_cortical
    Median_density_cancellous BMI
  /CRITERIA CILEVEL=95 CITYPE=PERCENTILE  NSAMPLES=1000
  /MISSING USERMISSING=EXCLUDE.
EXAMINE VARIABLES=Age Body_height Soft_tissue_thickness MSB_height
MSB_thickness_thickest
    MSB_thickness_thinnest MSB_width_thickest MSB_width_thinnest Median_density_cortical
    Median_density_cancellous BMI
  /PLOT NONE
  /STATISTICS DESCRIPTIVES
  /CINTERVAL 95
  /MISSING LISTWISE
  /NOTOTAL.
*Morphometry vs. Sex & Body height
CORRELATIONS
  /VARIABLES=MSB_height Body_height Male
  /PRINT=TWOTAIL NOSIG FULL
  /MISSING=PAIRWISE.


GRAPH
  /SCATTERPLOT(BIVAR)=Body_height WITH MSB_height
  /MISSING=LISTWISE.
REGRESSION
  /MISSING LISTWISE
  /STATISTICS COEFF OUTS CI(95) R ANOVA CHANGE
  /CRITERIA=PIN(.05) POUT(.10)
  /NOORIGIN
```

```
  /DEPENDENT MSB_height
  /METHOD=ENTER Male
  /SCATTERPLOT=(*ZRESID ,*ZPRED)
  /RESIDUALS NORMPROB(ZRESID).
REGRESSION
  /MISSING LISTWISE
  /STATISTICS COEFF OUTS CI(95) R ANOVA CHANGE
  /CRITERIA=PIN(.05) POUT(.10)
  /NOORIGIN
  /DEPENDENT MSB_height
  /METHOD=ENTER Body_height Male
  /SCATTERPLOT=(*ZRESID ,*ZPRED)
  /RESIDUALS NORMPROB(ZRESID).
CORRELATIONS
  /VARIABLES=MSB_thickness_thickest Male Body_height
  /PRINT=TWOTAIL NOSIG FULL
  /MISSING=PAIRWISE.
GRAPH
  /SCATTERPLOT(BIVAR)=Body_height WITH MSB_thickness_thickest
  /MISSING=LISTWISE.

REGRESSION
  /MISSING LISTWISE
  /STATISTICS COEFF OUTS CI(95) R ANOVA CHANGE
  /CRITERIA=PIN(.05) POUT(.10)
  /NOORIGIN
  /DEPENDENT MSB_thickness_thickest
  /METHOD=ENTER Male
  /SCATTERPLOT=(*ZRESID ,*ZPRED)
  /RESIDUALS NORMPROB(ZRESID).
REGRESSION
  /MISSING LISTWISE
  /STATISTICS COEFF OUTS CI(95) R ANOVA CHANGE
  /CRITERIA=PIN(.05) POUT(.10)
```

```
  /NOORIGIN
  /DEPENDENT MSB_thickness_thickest
  /METHOD=ENTER Body_height Male
  /SCATTERPLOT=(*ZRESID ,*ZPRED)
  /RESIDUALS NORMPROB(ZRESID).
CORRELATIONS
  /VARIABLES=MSB_thickness_thinnest Body_height Male
  /PRINT=TWOTAIL NOSIG FULL
  /MISSING=PAIRWISE.
GRAPH
  /SCATTERPLOT(BIVAR)=Body_height WITH MSB_thickness_thinnest
  /MISSING=LISTWISE.
REGRESSION
  /MISSING LISTWISE
  /STATISTICS COEFF OUTS CI(95) R ANOVA CHANGE
  /CRITERIA=PIN(.05) POUT(.10)
  /NOORIGIN
  /DEPENDENT MSB_thickness_thinnest
  /METHOD=ENTER Male
  /SCATTERPLOT=(*ZRESID ,*ZPRED)
  /RESIDUALS NORMPROB(ZRESID).
REGRESSION
  /MISSING LISTWISE
  /STATISTICS COEFF OUTS CI(95) R ANOVA CHANGE
  /CRITERIA=PIN(.05) POUT(.10)
  /NOORIGIN
  /DEPENDENT MSB_thickness_thinnest
  /METHOD=ENTER Body_height Male
  /SCATTERPLOT=(*ZRESID ,*ZPRED)
  /RESIDUALS NORMPROB(ZRESID).
CORRELATIONS
  /VARIABLES=MSB_width_thickest Body_height Male
  /PRINT=TWOTAIL NOSIG FULL
  /MISSING=PAIRWISE.
```

```
GRAPH
  /SCATTERPLOT(BIVAR)=Body_height WITH MSB_width_thickest
  /MISSING=LISTWISE.
REGRESSION
  /MISSING LISTWISE
  /STATISTICS COEFF OUTS CI(95) R ANOVA CHANGE
  /CRITERIA=PIN(.05) POUT(.10)
  /NOORIGIN
  /DEPENDENT MSB_width_thickest
  /METHOD=ENTER Male
  /SCATTERPLOT=(*ZRESID ,*ZPRED)
  /RESIDUALS NORMPROB(ZRESID).
REGRESSION
  /MISSING LISTWISE
  /STATISTICS COEFF OUTS CI(95) R ANOVA CHANGE
  /CRITERIA=PIN(.05) POUT(.10)
  /NOORIGIN
  /DEPENDENT MSB_width_thickest
  /METHOD=ENTER Body_height Male
  /SCATTERPLOT=(*ZRESID ,*ZPRED)
  /RESIDUALS NORMPROB(ZRESID).
CORRELATIONS
  /VARIABLES=MSB_width_thinnest Body_height Male
  /PRINT=TWOTAIL NOSIG FULL
  /MISSING=PAIRWISE.
GRAPH
  /SCATTERPLOT(BIVAR)=Body_height WITH MSB_width_thinnest
  /MISSING=LISTWISE.
REGRESSION
  /MISSING LISTWISE
  /STATISTICS COEFF OUTS CI(95) R ANOVA CHANGE
  /CRITERIA=PIN(.05) POUT(.10)
  /NOORIGIN
  /DEPENDENT MSB_width_thinnest
```

```
  /METHOD=ENTER Male
  /SCATTERPLOT=(*ZRESID ,*ZPRED)
  /RESIDUALS NORMPROB(ZRESID).
REGRESSION
  /MISSING LISTWISE
  /STATISTICS COEFF OUTS CI(95) R ANOVA CHANGE
  /CRITERIA=PIN(.05) POUT(.10)
  /NOORIGIN
  /DEPENDENT MSB_width_thinnest
  /METHOD=ENTER Body_height Male
  /SCATTERPLOT=(*ZRESID ,*ZPRED)
  /RESIDUALS NORMPROB(ZRESID).
*Bone density vs. Sex, Age & Smoking status
CORRELATIONS
  /VARIABLES=Median_density_cortical Smoking_status_past Smoking_status_current Age Male
  /PRINT=TWOTAIL NOSIG FULL
  /MISSING=PAIRWISE.
GRAPH
  /SCATTERPLOT(BIVAR)=Age WITH Median_density_cortical
  /MISSING=LISTWISE.
REGRESSION
  /MISSING LISTWISE
  /STATISTICS COEFF OUTS CI(95) R ANOVA CHANGE
  /CRITERIA=PIN(.05) POUT(.10)
  /NOORIGIN
  /DEPENDENT Median_density_cortical
  /METHOD=ENTER Age
  /SCATTERPLOT=(*ZRESID ,*ZPRED)
  /RESIDUALS NORMPROB(ZRESID).
REGRESSION
  /MISSING LISTWISE
  /STATISTICS COEFF OUTS CI(95) R ANOVA CHANGE
  /CRITERIA=PIN(.05) POUT(.10)
  /NOORIGIN
```

```
  /DEPENDENT Median_density_cortical
  /METHOD=ENTER Male
  /SCATTERPLOT=(*ZRESID ,*ZPRED)
  /RESIDUALS NORMPROB(ZRESID).
REGRESSION
  /MISSING LISTWISE
  /STATISTICS COEFF OUTS CI(95) R ANOVA CHANGE
  /CRITERIA=PIN(.05) POUT(.10)
  /NOORIGIN
  /DEPENDENT Median_density_cortical
  /METHOD=ENTER Smoking_status_past Smoking_status_current Age Male
  /SCATTERPLOT=(*ZRESID ,*ZPRED)
  /RESIDUALS NORMPROB(ZRESID).
CORRELATIONS
  /VARIABLES=Median_density_cancellous Smoking_status_past Smoking_status_current Age Male
  /PRINT=TWOTAIL NOSIG FULL
  /MISSING=PAIRWISE.
GRAPH
  /SCATTERPLOT(BIVAR)=Age WITH Median_density_cancellous
  /MISSING=LISTWISE.
REGRESSION
  /MISSING LISTWISE
  /STATISTICS COEFF OUTS CI(95) R ANOVA CHANGE
  /CRITERIA=PIN(.05) POUT(.10)
  /NOORIGIN
  /DEPENDENT Median_density_cancellous
  /METHOD=ENTER Age
  /SCATTERPLOT=(*ZRESID ,*ZPRED)
  /RESIDUALS NORMPROB(ZRESID).
REGRESSION
  /MISSING LISTWISE
  /STATISTICS COEFF OUTS CI(95) R ANOVA CHANGE
  /CRITERIA=PIN(.05) POUT(.10)
  /NOORIGIN
```

```
  /DEPENDENT Median_density_cancellous
  /METHOD=ENTER Male
  /SCATTERPLOT=(*ZRESID ,*ZPRED)
  /RESIDUALS NORMPROB(ZRESID).
REGRESSION
  /MISSING LISTWISE
  /STATISTICS COEFF OUTS CI(95) R ANOVA CHANGE
  /CRITERIA=PIN(.05) POUT(.10)
  /NOORIGIN
  /DEPENDENT Median_density_cancellous
  /METHOD=ENTER Smoking_status_past Smoking_status_current Age Male
  /SCATTERPLOT=(*ZRESID ,*ZPRED)
  /RESIDUALS NORMPROB(ZRESID).
*BMI vs. Soft tissue thickness
CORRELATIONS
  /VARIABLES=Soft_tissue_thickness BMI
  /PRINT=TWOTAIL NOSIG FULL
  /MISSING=PAIRWISE.
GRAPH
  /SCATTERPLOT(BIVAR)=BMI WITH Soft_tissue_thickness
  /MISSING=LISTWISE.
REGRESSION
  /MISSING LISTWISE
  /STATISTICS COEFF OUTS CI(95) R ANOVA CHANGE
  /CRITERIA=PIN(.05) POUT(.10)
  /NOORIGIN
  /DEPENDENT Soft_tissue_thickness
  /METHOD=ENTER BMI
  /SCATTERPLOT=(*ZRESID ,*ZPRED)
  /RESIDUALS NORMPROB(ZRESID).
```

Proposed scanning protocol

Patient preparation

Fasting: NA

Remove metal: especially necklaces/chains/chokers/etc.

Intravenous access: NA

Scan parameters

Parameter	Value
Scanner type	Multidetector CT (64-slice or higher)
Scan range	First cervical vertebra to seventh thoracic vertebra (C1–T7)
Slice thickness	0.5–1.25 mm (thin slices for 3D recon)
Reconstruction	Axial, coronal, sagittal (1–3 mm thick)
Field of view	Small, focused on area of interest
Pitch	0.9–1.2
Voltage	100–120 kVp
Amperage	Depending on dose
Kernel	Bone tissue

Body positioning

Positioning: Supine, head first, arms by side, as flat as possible: legs stretched straight, with the head in extension on the pillow. It is important that the patient lies symmetrically on the table, so the cervical and thoracic spine should, as much as possible, be in line with the sagittal axis, as should the manubrium sterni bone. The scout view can be checked to determine symmetry before scanning commences.
